# Ultrasmall Nanogels from Intramolecular Cross-Linking of (Hyper)Branched Cyclic Polyglycerols with Diboronic Acids

**DOI:** 10.1021/acs.macromol.5c02780

**Published:** 2026-03-30

**Authors:** Eric Gómez Urreizti, Paula Malo de Molina, Carlo Andrea Pagnacco, Jose A. Pomposo, Reidar Lund, Fabienne Barroso-Bujans

**Affiliations:** a 226245 Donostia International Physics Center (DIPC), Paseo Manuel Lardizábal 4, 20018 Donostia−San Sebastián, Spain; b Centro de Física de Materiales (CSIC-UPV/EHU), Materials Physics Center MPC, Paseo Manuel Lardizábal 5, 20018 Donostia−San Sebastián, Spain; c PMAS, Faculty of Chemistry, University of the Basque Country (UPV/EHU), Paseo Manuel Lardizábal 3, 20018 Donostia−San Sebastián, Spain; d IKERBASQUE, Basque Foundation for Science, Plaza Euskadi 5, 48009 Bilbao, Spain; e Department of Chemistry, 6305 University of Oslo, Postboks 1033, Blindern, Oslo 0315, Norway; f Hylleraas Centre for Quantum Molecular Sciences, University of Oslo, Postboks 1033, Blindern, Oslo 0315, Norway

## Abstract

We report the synthesis of ultrasmall nanogels (*R*_g_ < 6 nm) from branched and hyperbranched cyclic polyglycerols (*M*_n_ = 3–200 kg/mol) via intramolecular cross-linking with diboronic acids (benzene-1,4-diboronic acid, Bz-2B, and [(1,2-diphenylethene-1,2-diyl)­bis­(4,1-phenylene)] diboronic acid, TPE-2B) through dynamic covalent bonding. Structural analysis by small-angle X-ray scattering revealed a clear architecture–conformation relationship: high-molecular-weight hyperbranched cyclic polyglycerols formed compact, spherical structures, whereas low-molecular-weight branched analogues displayed open, fractal-like structures. Cross-linking preserved the overall size and conformation of the precursors, providing strong evidence of predominantly intramolecular nanogel formation with negligible aggregation, as indicated by the minimal low-*Q* scattering contribution. Incorporation of TPE-2B enabled fluorescence probing of nanogel structuring, revealing architecture-dependent localization of the fluorogen at the polymer periphery or within the interior. Enhanced emission arose from restricted intramolecular rotation of the phenyl rotors and was governed by the molecular weight of the polymer scaffold and the degree of fluorogen confinement. Nanogels formed with Bz-2B were able to physically entrap rhodamine B within their interstitial spaces, exhibiting a release behavior modulated by cross-linking density and molecular weight. These findings establish an architecture-dependent class of ultrasmall nanogels and demonstrate how dynamic covalent cross-linking, combined with fluorescence probing and scattering analysis, provides detailed insight into structure–property relationships in confined polymer nanostructures.

## Graphical abstract

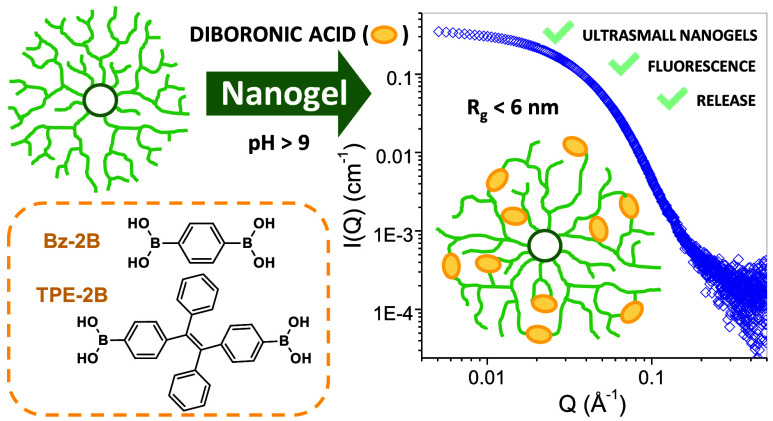

## Introduction

Nanogels are composed of cross-linked polymer networks that can swell in water or biological fluids, making them highly versatile for various applications, especially in biomedicine.^1–3^ These three-dimensional structures are typically smaller than 100 nm. When the size is in the micrometer range, they are called microgels.^4^ However, submicrometer structures are sometimes also referred to as microgels.^5,6^ Nanogels have gained significant attention in drug delivery because of their ability to encapsulate and protect a wide range of therapeutic agents, including hydrophobic drugs, proteins, and nucleic acids, while providing controlled and targeted release through stimuli-responsive mechanisms.^1–3,6^

Nanogels can be obtained through two main approaches: (i) polymerization of monomers in the presence of a cross-linker, typically carried out in dispersed systems such as precipitation, suspension, or emulsion polymerization; (ii) intramolecular or intermolecular cross-linking of preformed polymer chains under high dilution or within confined environments such as micelles, miniemulsions, or microfluidic systems.^1–3,5,6^

A special class of nanogels is formed from hyperbranched (hb) polyglycerol (PG) (hbPG). These nanogels result from cross-linking the branches, resulting in stable and compact nanogel structures with advantages such as uniform size, high surface functionality, and controlled porosity. Haag et al.^7^ used the miniemulsion polymerization technique to cross-link hbPG structures and form hbPG clusters, also called “megamers”,^8^ with variable diameters from 25 to 90 nm. In another approach, Haag et al.^9^ synthesized intramolecularly cross-linked polyglycerol nanoparticles with diameters up to 80 nm by recourse to cationic copolymerization of glycerol and triglycidylglycerol catalyzed by *p*-toluenesulfonic acid under miniemulsion conditions. Zhou et al.^10^ produced hbPG-based nanogels of controllable diameter of 90–400 nm by UV light irradiation of thiolated hbPGs, which spontaneously self-assembled in water through hydrophobic interactions. Hedtrich et al.^11^ prepared protein-loaded thermoresponsive poly­(*N*-isopropylacrylamide)-hbPG-based nanogels of about 200 nm diameter and demonstrated their potential application for cutaneous protein delivery.

By contrast, only a few studies have explored intramolecular cross-linking of preformed hbPGs, leading to structures that maintain their single-molecule size. One approach was the ring-closing metathesis of allyl groups located either on the PG surface to generate cross-linked architectures with different loop-sizes,^12,13^ or throughout the PG structure.^14^ The closed-shell hbPG hosts were able to transport organic dyes by demonstrating that their uptake capability was strongly dependent on the loop size, while maintaining their single-molecule hydrodynamic volume intact.^12^ A cross-linking approach over the entire hbPG structure has been used to encapsulate organic dyes (e.g., fluorescein and perylene diimide) and demonstrate improved photophysical behavior of the dyes.^14^ However, although cross-linking was successful, similar results were obtained with the non-cross-linked hbPG structures, which also showed improved photostability over free fluorescein and transport capability for perylene diimide in water.

Here, we introduce a new concept: hbPG nanogels formed by intramolecular dynamic covalent cross-linking with boronic acids, inspired by extensive work on single-chain polymer nanoparticles (SCNPs). A large number of reactions, irreversible and reversible, have been used to prepare the so-called SCNPs upon the collapse of linear polymer chains.^15–21^ While irreversible reactions, such as alkene metathesis and click cycloaddition, have been most commonly used in the intramolecular cross-linking of dendrimers, the difficulties associated with the synthesis of dendrimers and the lower chemical diversity compared to linear polymers would probably explain the lower number of studies on cross-linked dendrimers compared to SCNPs. Alternatively, hbPGs,^22^ like branched cyclic PGs (bcPGs),^23,24^ are structures analogous to dendrimers and are relatively easy to synthesize in a one-step process. Their intramolecular cross-linking has been barely addressed, with the exceptions given above for hbPGs.^9,12–14^

Boronic acids provide a promising opportunity to address this gap. Beyond serving as efficient cross-linkers, they bring great versatility due to the wide structural diversity of diboronic acids (varying in length, rigidity, and functional substitution) which enables fine-tuning of network architecture and the introduction of additional functionalities.^25,26^ The ability of boronic acids to form reversible covalent bonds with diols and Lewis bases makes them invaluable in molecular recognition and sensing, particularly in glucose biosensors.^27,28^ In the field of vitrimeric^29,30^ and “self-healing” materials,^31^ boronic acids and their ester derivatives enable the creation of polymer networks that can autonomously repair damage, thereby increasing durability. In addition, boronic acids are integral to advanced drug delivery systems,^26,32^ where they facilitate responsive release mechanisms triggered by specific biological conditions. Their utility extends to catalysis,^33^ micellar stabilization,^34^ surface modification,^35^ where they impart tailored functionalities to materials.

To obtain well-defined ultrasmall nanogels, it is essential to start from polymer precursors with a controlled architecture and functionality. bcPGs consist of a cyclic core decorated with randomly distributed branches and therefore share key structural features with multihydroxylic hbPGs, while differing in their degree of compaction and core geometry.^36^ Whereas hbPGs are typically synthesized from a trihydroxyl initiator and adopt compact, globular conformations,^37^ bcPGs exhibit a more open architecture with looser branches.^36,38^ In this work, bcPGs are therefore employed as a versatile and synthetically accessible platform to systematically evaluate nanogel formation across a wide range of molecular weights and branching densities while establishing a general synthetic route that is equally applicable to hbPGs.

The electrophilic zwitterionic ring expansion polymerization (eZREP) of glycidol (Gly) with B­(C_6_F_5_)_3_ is an efficient method for the synthesis of bcPG in one pot.^23,24^ However, the maximum molecular weight obtained under bulk conditions is about 10 kg/mol.^23^ In solution, the molecular weight does not increase much either.^39^ The polymerization in toluene occurs with the formation of two phases from the very beginning of the polymerization.^39^ Chain growth continues in the precipitated phase, similar to bulk polymerization. Chain fusion events are thought to be responsible for the increase in molecular weight at very low content of free monomer.^23,39^ In the present study, we used a combination of eZREP and ring-opening multibranching polymerization (ROMBP) to generate high-molecular-weight hyperbranched cyclic polyglycerols (hbcPG) with molecular weights of up to 200 kg/mol. The term hyperbranched refers to a very high degree of branching (DB) typically obtained by ROMBP of Gly with values of >0.5,^22^ in contrast to the lower DB obtained by eZREP with values of <0.5.^23,24^

In this study, we evaluate the formation of intramolecular cross-linked (h)­bcPG structures using the boronic acids described below:

(1) Benzene-1,4-diboronic acid (Bz-2B) is a simple molecule used to create cross-linked polymer structures, e.g., SCNPs,^20^ vitrimers,^30^ and hydrogels.^40^ Its monoboronic acid analogue has been widely used to evaluate the underling mechanisms behind the acid–base equilibrium in the formation of boronic esters and the determination of association constants.^41^
(2) [(1,2-Diphenylethene-1,2-diyl)­bis­(4,1-phenylene)] diboronic acid (TPE-2B) has the property of emitting light when aggregated and in the solid state but does not emit in dilute solution, exhibiting an aggregation-induced emission (AIE) effect.^27^ The main cause of the AIE has been attributed to the restriction of intramolecular rotation (RIR) of phenyl rotors.^42^ TPE-2B is capable of reacting with d-glucose at high pH by exhibiting AIE enhanced by RIR.^27^ The formation of oligomeric rigid structures between TPE-2B and d-glucose is thought to be responsible for its intense emission, in contrast to other saccharide molecules containing only one cis-diol group (d-fructose, d-galactose, and d-manose), which do not oligomerize. In a more general concept, molecules that are AIE-active are being widely explored in the field of biomedicine due to the advantages that fluorescence imaging brings.^43^

Ultrasmall nanogels were generated through the intramolecular cross-linking of bcPG and hbcPG by using Bz-2B and TPE-2B. The structure of the nanogels was characterized by small-angle X-ray scattering (SAXS) in aqueous solutions under basic pH conditions, where cross-linking is promoted through boronic ester formation. The results evidenced the formation of ultrasmall nanogels with radii of gyration (*R*_g_) ranging from 2 to 6 nm with predominantly intramolecular cross-linking and no measurable particle growth or large-scale aggregation. Thanks to the RIR effect in TPE-2B molecules, fluorescence spectroscopy revealed additional structural features in these nanogels: TPE-2B is primarily located on the periphery of low-molecular-weight bcPG (3 and 10 kg/mol), but it is internalized within high-molecular-weight hbcPG (60 and 200 kg/mol) and emits light up to ∼50 times more than free TPE-2B. Moreover, the entrapment of rhodamine B (RhB) within these ultrasmall nanogels, followed by its slow release, demonstrated their ability to encapsulate and retain small molecular probes, providing insight into how dynamic intramolecular cross-linking governs molecular confinement and transport within ultrasmall polymer nanostructures.

## Materials and Methods

### Materials

Glycidol (Gly) and toluene were distilled from CaH_2_ under reduced pressure, and B­(C_6_F_5_)_3_ was sublimated. Sodium hydride (NaH), sodium bicarbonate (NaHCO_3_), sodium carbonate (Na_2_CO_3)_, dichloromethane, dimethyl sulfoxide, methanol, diethyl ether, and rhodamine B (RhB) were purchased from Sigma-Aldrich and used as received. Benzene-1,4-diboronic acid (Bz-2B) and [(1,2-diphenylethene-1,2-diyl)­bis­(4,1-phenylene)] diboronic acid (TPE-2B) were purchased from BLD pharm and used as received. A Na_2_CO_3_/NaHCO_3_ buffer was prepared, and the pH was adjusted to pH = 9 using NaOH (0.1 M).

### Synthesis of Branched Cyclic Polyglycidol (bcPG)

The reaction was performed in a jacketed three-necked flask equipped with a magnetic stirrer under an argon atmosphere as previously reported.^39^ A flask containing 3 mL of Gly (45.23 mmol) and 8 mL of toluene was cooled to −20 °C, and then 30 mg of B­(C_6_F_5_)_3_ (0.058 mmol) dissolved in 3 mL of toluene was added after temperature stabilization. The catalyst was also cooled to −20 °C before being added to the flask. The reaction was quenched after 24 h by adding 2 drops of DMF. Since two phases are formed during polymerization,^39^ the upper phase (liquid toluene phase) was separated from the lower phase (bulky gel-like phase) containing the polymer. This bulk phase was dissolved in MeOH and passed through basic Al_2_O_3_ to remove the residual catalyst from the solution. Finally, the polymer was recovered from the solution by using a rotary evaporator. A fraction of bcPG was obtained by fractional precipitation from a methanol solution (0.1 g/mL) with the addition of diethyl ether, followed by centrifugation and drying in a vacuum oven at 80 °C overnight and 120 °C for 1 h. Yield (wt %) = 53.

### Synthesis of Hyperbranched Cyclic Polyglycidol (hbcPG)

hbcPGs were synthesized in two steps. Step 1: A bcPG sample was first obtained using the same reaction conditions as described above, with the difference that the reaction was quenched after 5 min by adding 2 drops of DMF. The lower phase (bulky gel-like phase) containing the polymer was then dissolved in MeOH and passed through basic Al_2_O_3_. The polymer was recovered from the solution by using a rotary evaporator, dissolved in water and dialyzed using a 1 kDa MWCO membrane. Yield (wt %) = 69. Step 2: A round-bottomed flask was dried with a hot gun (600 °C) and filled with Ar. As a typical reaction for generating hbcPG of *M*_n_ ∼ 60 kg/mol, 50 mg of previously synthesized bcPG was added to the flask in a glovebox together with 10 mol % of NaH with respect to the total hydroxyl groups of bcPG. Then 300 μL of anhydrous DMSO was added under Ar flux. This mixture was stirred at 90 °C for 90 min, and a color change to brown was observed. Then, 1.56 mL of Gly was added dropwise with a syringe pump at 0.04 mL/h for 40 h at 90 °C. 1.35 mL of HCl (0.1 N in MeOH) was added as a quencher. The product was dissolved in water and dialyzed through a 6 kDa MWCO membrane. Water was removed using a rotary evaporator, and the polymer was dried in a vacuum oven at 80 °C for 4 h and then at 120 °C for 1 h. Yield (wt %) = 50.

### Synthesis of Nanogels (NGs) Cross-Linked with Bz-2B (NG:Bz-2B)

NG:Bz-2B nanogels were prepared at pH 9, using either a carbonate buffer or NaOH, as indicated for each experiment. To demonstrate the reversibility of the reaction in DOSY NMR experiments at pH 9 and pH 7, D_2_O solutions of the polymer (2 mg/mL) and Bz-2B (1 mg/mL, 6 mM) were first prepared independently. The pH of both solutions was then adjusted to pH = 9 using NaOH (1 M). 5 mL of the polymer solution was added to 2.8 mL of the Bz-2B solution overnight at room temperature using a syringe pump at a rate of 0.05 mL/h. Finally, the pH was adjusted to 7 with HCl (0.1 M). To evaluate the structure and chain conformation of the nanogels in SAXS experiments, NG:Bz-2B nanogels were prepared from polymers of different molecular weights. Bz-2B was first dissolved in pure DMSO and subsequently diluted with carbonate buffer to obtain a final concentration of 1.12 mg/mL in a solution containing 10% DMSO. DMSO was used to ensure complete dissolution of Bz-2B, which exhibits limited solubility in purely aqueous buffers at the concentrations required for the stock solution. The presence of DMSO additionally ensures that Bz-2B remains molecularly dispersed during the slow-addition process, which is important to favor intramolecular cross-linking over intermolecular aggregation. The polymer was dissolved separately at a concentration of 4 mg/mL in a carbonate buffer. Then, 3 mL of Bz-2B was added at a rate of 0.04 mL/h over 3 mL of the polymer solution. The resulting NG:Bz-2B was diluted to generate four distinct polymer concentrations ranging from 2 to 1 mg/mL. As a reference, polymer solutions were prepared at an identical concentration range in the carbonate buffer with 5% DMSO.

### Synthesis of Nanogels Cross-Linked with TPE-2B (NG:TPE-2B)

4 mL of a TPE-2B solution in DMSO (1.05 mg/mL) was added to 96 mL of carbonate buffer, yielding a 0.1 mM TPE-2B solution containing 4% DMSO. DMSO was used to ensure complete dissolution and molecular dispersion of TPE-2B, as in the previous case. In parallel, polymer solutions with concentrations of 6, 2, 1, 0.2, 0.02, 0.002, and 0.0002 mg/mL were prepared in carbonate buffer. 1 mL of the TPE-2B solution was then added to 1 mL of each polymer solution at a rate of 0.2 mL/h to obtain nanogels containing half the concentration of each mixture component. The fluorescence of all samples was measured under identical conditions. For the SAXS experiments, NG:TPE-2B samples were prepared at a final concentration of 3 mg/mL of polymer and 0.05 mM of TPE-2B in carbonate buffer containing 5% DMSO. The nanogels were subsequently diluted to obtain four distinct concentrations ranging from 3 to 1.5 mg/mL. Note that the same concentration of DMSO was used as in previous SAXS experiments for NG:Bz-2B samples.

### Release of Rhodamine B (RhB)

A designed U-shaped glass was used to conduct the experiment (Figure 1). The tube consists of two compartments separated by a 6 kDa MWCO dialysis membrane of 1 cm diameter, which was previously hydrated for 16 h in a carbonate buffer, pH 9. The sample solution containing RhB (4 mL) was added to zone [S] while carbonate buffer (10 mL) was added to zone [M]. To monitor the passage of RhB from zone [S] to [M], 1 mL aliquot of zone [M] was extracted and analyzed by UV–vis, returning the solution to the tube immediately after each measurement. A magnetic stirrer was used to ensure homogeneity of the solution. Peak maxima at 554 nm of the aliquots were normalized to that of a RhB solution prepared by adding 4 mL RhB (10 μg/mL) and 10 mL of carbonate buffer, pH 9, allowing the absorbance data to be expressed as % absorption. The nanogels were prepared as follows. 3 mL of hbcPG (48 μg/mL) and 3 mL of RhB (40 μg/mL) were mixed in a carbonate buffer, pH = 9, for 8 h. Then, 5 mL of the RhB/hbcPG mixture was added dropwise to 5 mL of buffered Bz-2B solutions (10.5, 7.0, and 5.2 μg/mL). A 10 μg/mL RhB solution was used for control experiments. The final polymer concentration was maintained at 12 μg/mL for all experiments.

**Figure 1:**
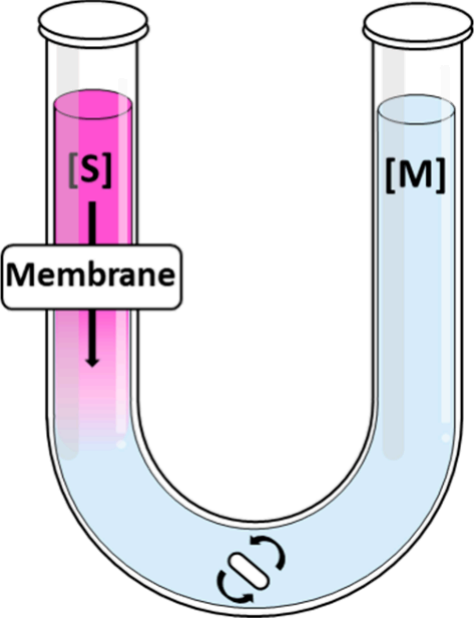
“U” shaped glass used to monitor the release of RhB from the nanogels.

### Characterization

#### NMR Spectroscopy

NMR spectra were recorded on a Bruker Avance Neo 500. Relative abundance of structural units and degree of branching (DB) of polyglycerols were determined by inverse-gated ^13^C NMR spectra following established procedures.^23^ Detailed peak assignments and integration schemes are provided in the Supporting Information. Around 30 mg of purified polymers was dissolved in 0.5 mL of D_2_O. The degree of branching (DB) was calculated from DB = 2*D*/(2*D* + *L*), where *D* and *L* (*L* = *L*_1,3_ + *L*_1,4_) are the relative abundance of dendritic and linear structures, respectively.^22^

#### Gel Permeation Chromatography (GPC)

GPC data were acquired using a Nexera instrument from Shimadzu using a refractive index detector (RID-20A, Shimadzu) and MALS detector (λ = 663.89 nm, miniDawn, Wyatt). DMF containing 0.1% LiBr with a flow of 1.0 mL/min was used as an eluent. Separation was performed at 50 °C by using a CTO 40C column oven and Polargel-M Guard 50 mm × 7.5 mm and Polargel-M 300 mm × 7.5 mm, 8 μm, GPC columns. The absolute molecular weights were determined using a d*n*/d*c* value^44^ of 0.054 mL/g and Astra 8.1 software from Wyatt Technology.

#### UV–vis Spectroscopy

UV–vis spectra of RhB solutions were recorded in an Agilent 8453A with a Peltier thermostated cell holder, T-controller 89090A, using a quartz cuvette of 1 cm path length.

#### Fluorescence Spectroscopy

Fluorescence spectra were recorded using a Synergy H1 microplate reader (BioTek Instruments, Winooski, VT, USA) controlled by Gen5 software. Samples were excited at 365 nm, and emission spectra were recorded from 375 to 700 nm with a 1 nm increment. The “Sweep” read mode was used with a delay of 100 ms.

#### Dynamic Light Scattering (DLS)

A 3D-LS spectrometer (LS Instruments AG, Fribourg, Switzerland) was used to characterize the structural conformation of the samples. The instrument is equipped with a high-performance 100 mW DPSS laser operating at a wavelength of λ = 660 nm. Samples were placed in cylindrical glass cuvettes with a diameter of 1 cm and positioned inside an index-matching vat filled with decalin at 25 °C. The magnitude of the scattering vector was calculated as *Q* = 4π*n*_s_ sin­(θ/2)/λ where *n*_s_ is the refractive index of the solvent. Hydrodynamic radii were obtained from second-order cumulant analysis of the autocorrelation function.^45^

#### Small-Angle X-ray Scattering (SAXS)

The SAXS experiments were performed using the automated BM29 bioSAXS beamline at the ESRF, Grenoble, France. For technical details we refer to ref 46. The data were obtained using an energy of 12.5 keV and detector distance 2.87 m covering a *Q*-range (*Q* = 4π sin­(θ/2)/λ, where λ is the wavelength, θ is the scattering angle) of about 0.0047 Å^–1^ < *Q* < 0.5 Å^–1^. The data were calibrated to an absolute intensity scale using water as a primary standard.

#### Theoretical Modeling of SAXS Data

To model the scattering from the polymer solutions, we used two different approaches. First the fractal polymer like structures at low molecular weights were described using the generic generalized Debye model introduced by Hammouda:^47^

1 $${P\left(Q\right)}_{\mathrm{poly}}=\frac{1}{\nu \cdot U^{1/\left(2\nu \right)}}\Gamma_{\mathrm{inc}}\left(\frac{1}{2\nu },U\right)-\frac{1}{\nu \cdot U^{1/\nu }}\Gamma_{\mathrm{inc}}\left(\frac{1}{\nu },U\right)$$

where *U* = $\frac{{\left(QR_{g}\right)}^{2}\left(2\nu +2\right)\left(2\nu +1\right)}{6},$ ν is the Flory exponent related to the fractal dimension as *d*_f_ = 1/ν and Γ_inc_(*x*,*U*) = ∫_0_^*U*^ exp (−*t*)*t* ^*x*–1^ d*t* is the incomplete gamma function and *R*_g_ is the radius of gyration.

For high molecular weight hyperbranched polymers, which have more compact structures, we used a “fuzzy sphere” model to account for a potential inhomogeneous distribution within the nanoparticles. The scattering form factor, can then be calculated using a spherical form factor with a Debye–Waller factor taking into account a graded interface:^48^

2 $${P\left(Q,R_{p},\sigma_{Rp}\right)}_{\mathrm{fuzzy}}={\left[\frac{3\left[\sin\left(QR_{p}\right)-QR_{p}\cos\left(QR_{p}\right)\right]}{{\left(QR_{p}\right)}^{3}}\exp\left(-\frac{{\left(Q{\cdot \sigma }_{Rp}\right)}^{2}}{2}\right)\right]}^{2}$$

*R*_p_ is the overall size, and σ_*Rp*_ is the width of the interface. In order to take into account polydispersity of particle sizes, a Gaussian distribution was included and an average of the form factor was calculated according to

$${\left\langle P\left(Q\right)\right\rangle }_{\mathrm{fuzzy}}=,\int_{0}^{\infty }\frac{1}{\sigma_{p}\sqrt{2\pi }}\exp\left(-\frac{{\left(R_{p}-\left\langle R_{p}\right\rangle \right)}^{2}}{2{\sigma_{p}}^{2}}\right){P\left(Q,R_{p}\right)}_{\mathrm{fuzzy}}dR_{p}$$ 3

Since the nanoparticles are expected to be swollen with solvent, the internal polymer-like scattering contribution must be included. This can be included by adding a “blob scattering” term to the total scattering:

4 $$I\left(Q\right)=c/\left({d_{p}}^{2}N_{A}\right)\cdot M_{w}{\left(\rho_{P}-\rho_{0}\right)}^{2}{\left\langle P\left(Q\right)\right\rangle }_{\mathrm{fuzzy}}+{I\left(Q\right)}_{\mathrm{blob}}$$

where *c* is the concentration of the solute in mg/mL, *d* is the solute density, *M*_w_ is the weight-average molar mass, ρ_p_ and ρ_0_ are the scattering length density for the polymer and solvent, respectively. For the blob scattering describing internal chain segment correlation, we used the following well-known Ornstein–Zernike expression:

5 $${I\left(Q\right)}_{\mathrm{blob}}=\frac{\mathrm{Bc}}{{1+\left(Q\xi \right)}^{2}}$$

where ξ is the blob size and *B* is a scaling constant.

The solute density also required to estimate ρ_p_ was measured using an Anton Paar DMA5000 densitometer and gave *d*_p_ = 1.34 g/mL. The scattering length density is calculated according to

6 $$\rho_{p}=\frac{\underset{i}{\sum }Z_{i}}{\overset{®}{M_{0}/d_{p}\;}}r_{0}$$

where *M*_0_ is the molecular weight of the monomer and *r*_0_ is the Thomson radius. For the polyglycidol precursor (C_3_O_2_H_6_), we obtained ρ_p_ = 1.228 × 10^11^ cm^–2^. For the nanogels we calculated, assuming full insertion of the added cross-linkers, (C_3_O_2_H_6_)_0.89_­(C_6_B_2_O_2_H_2_)_0.11_, ρ_p_ = 1.217 × 10^11^ cm^–2^ in the case of Bz-2B and (C_3_O_2_H_6_)_0.99_­(C_26_H_22_B_2_O_4_)_0.01_, ρ_p_ = 1.231 × 10^11^ cm^–2^ for TPE-2B.

Finally, the molecular weight was obtained from Guinier analysis: *I*(*Q*) = *I*(0) exp­[−*R*_g_^2^ *Q* ^2^/3], with *M*_w_ = *I*(0)*d*_p_^2^ *N*_A_/[*c*(ρ_P_ – ρ_0_)^2^] using four different concentrations. The molecular weight was first estimated for polymer solutions in water. The results for *M*_*w*_ were used as a calibration to estimate the contrast for the buffered solutions in DMSO where pH adjustment with NaOH impeded accurate estimation.

## Results and Discussion

### Preparation of Nanogels by Cross-Linking (Hyper)­Branched Cyclic Polyglycerols with Benzene-1,4-diboronic Acid

The hbcPG structures were generated in two steps: eZREP and ROMBP (Figure 2). First step included the polymerization of Gly with B­(C_6_F_5_)_3_ for only 5 min to ensure the formation of low molecular weight bcPG structures according to our previous kinetic experiments.^39^ Then, a purified fraction of bcPG was dialyzed to remove any excess Gly, dried, and used as a macroinitiator for the synthesis of hbcPG (entry 1, Table 1). Hydroxyl groups were activated with NaH in DMSO followed by a slow addition of Gly. By varying the [Gly] to [bcPG] ratio, different molecular weights of hbcPGs were obtained (entries 3–5, Table 1). In those cases, the DB increased to values of >0.5. A bcPG reference structure was also synthesized by eZREP of Gly with B­(C_6_F_5_)_3_ for 24 h in a one-pot polymerization, which allowed to generate a sample with *M*_n_ = 10 kDa and DB 0.38 after precipitation (entry 2, Table 1), as expected from our previous studies.^39^ MALDI-ToF mass spectrometry data confirmed the formation of targeted bcPG structures (Figure S1).

**Figure 2:**
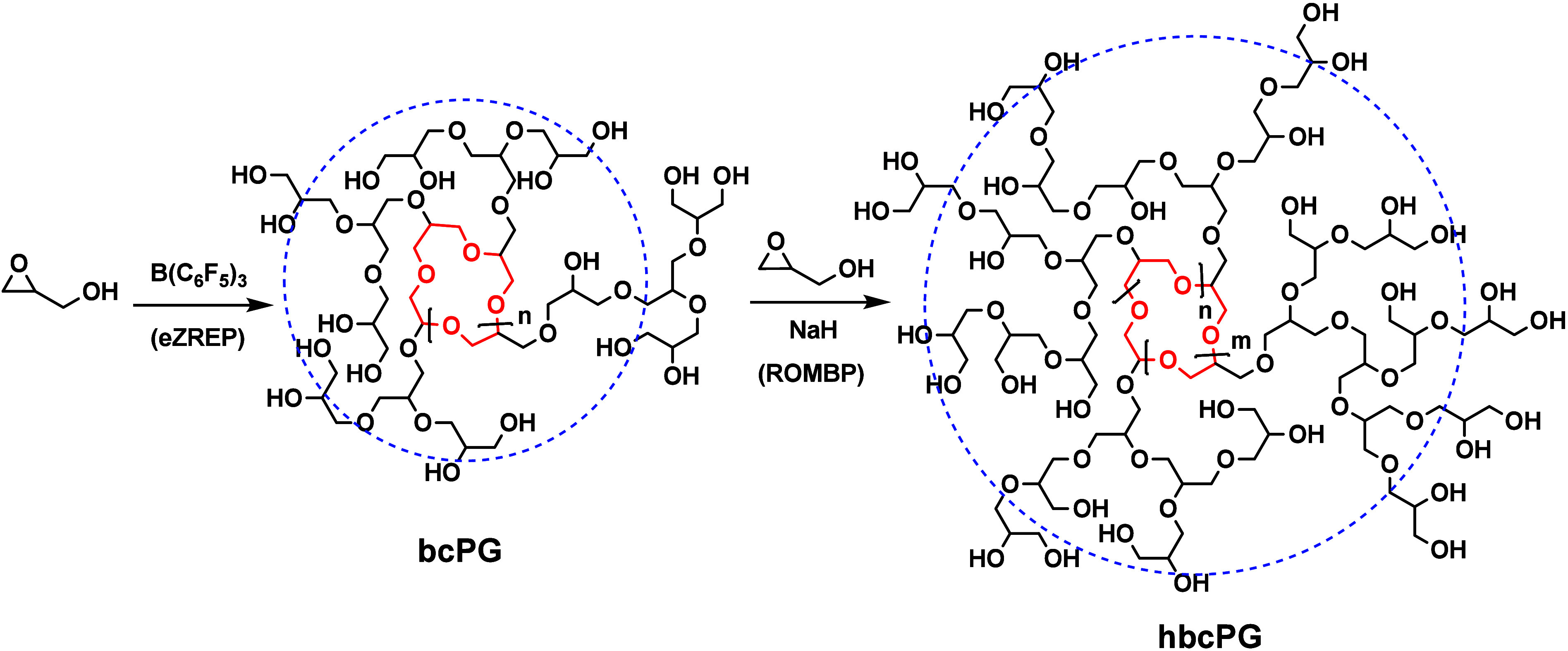
Multistep synthesis of high molecular weight hbcPG.

**Table 1:** Macromolecular characteristics of bcPG and hbcPG

				relative abundance (%)^b^									
entry	name	*M*_n_ ^a^ (kg/mol)	Đ	L_1,3_	D	L_1,4_	T_1_	T_2_	diol	DB	*R*_g_ (nm)^c^	*R*_h_ (nm)^d^	*R*_g_/*R*_h_
1	bcPG_3k_	3.8	1.5	33	17	24	19	7	31	0.37	1.91	2.1	0.90
2	bcPG_10k_	10.1	1.4	33	18	26	18	5	28	0.38	2.66	3.4	0.78
3	hbcPG_40k_	42.8	1.9	11	22	28	39	0	50	0.53	3.62	4.9	0.74
4	hbcPG_60k_	63.9	1.8	12	21	28	39	0	49	0.51	4.39	5.8	0.76
5	hbcPG_200k_	207.4	1.8	11	30	27	32	0	46	0.60	6.30	7.7	0.82

a Determined by GPC-RI-MALS in DMF (+LiBr 0.1%).

b Determined by inverse-gated ^13^C NMR (Figure S2). The relative diol content, defined as the fraction of diol groups with respect to the total hydroxyl functionalities in the sample, was estimated as (*T*_1_ + *T*_2_)/(*T*_1_ + *T*_2_ + *L*_1,3_ + *L*_1,4_).

c Determined by SAXS (Figure S4) using water as a solvent.

d Determined by DLS using water as a solvent.

To ensure predominantly intramolecular cross-linking, nanogel formation was carried out under deliberately dilute conditions, using polymer concentrations below 3 mg mL^–1^, controlled stoichiometries with molar equivalents of boronic acid relative to polymer diol functionalities ([boronic acid]/[diol]) below unity (see Table S1), and gradual addition of the predissolved polymer solution to a diboronic acid solution at pH 9. This combination of conditions and the order of addition was deliberately chosen to maintain the polymer under highly dilute conditions during the cross-linking reaction, thereby favoring intramolecular cross-linking within individual (h)­bcPG entities while minimizing intermolecular reactions and aggregation.

The reaction of diol moieties of polyglycerol structures with functional diboronic acids leads to pH-dependent reversible ester formation,^20,28,41^ as shown in Figure 3. ^1^H NMR confirmed the formation of boronic esters upon reaction of Bz-2B with bcPG_10k_ in a buffer at pH 9, as evidenced by a 0.3 ppm upfield shift and signal narrowing of the aromatic protons of Bz-2B (Figure 4a). This observation was further corroborated by analysis of a higher-molecular-weight nanogel obtained from hbcPG_60k_ and by reducing the [boronic acid]/[diol] molar equivalent ratio from 0.8 to 0.4 (Figure S3). Additionally, the sharp appearance of the a′ signal at 7.4 ppm and the absence of additional downfield resonances in all spectra suggest anchoring of the diboronic acid predominantly through diboronic ester formation. This behavior contrasts with a previous report on hydrogels formed by cross-linking hbPG with Bz-2B, which require much higher polymer concentrations despite comparable [boronic acid]/[diol] ratios exhibiting multiple ^1^H NMR signals arising from the formation of boronic monoesters and diesters.^49^ Owing to the high polymer dilution and the relatively low amount of boronic acid per diol unit in our experiments, quantitative determination of an absolute degree of cross-linking by ^1^H and ^11^B NMR is not straightforward. Consequently, the cross-linking efficiency is evaluated through its structural and functional consequences, as revealed by SAXS, fluorescence probing, and release kinetics.

**Figure 3:**
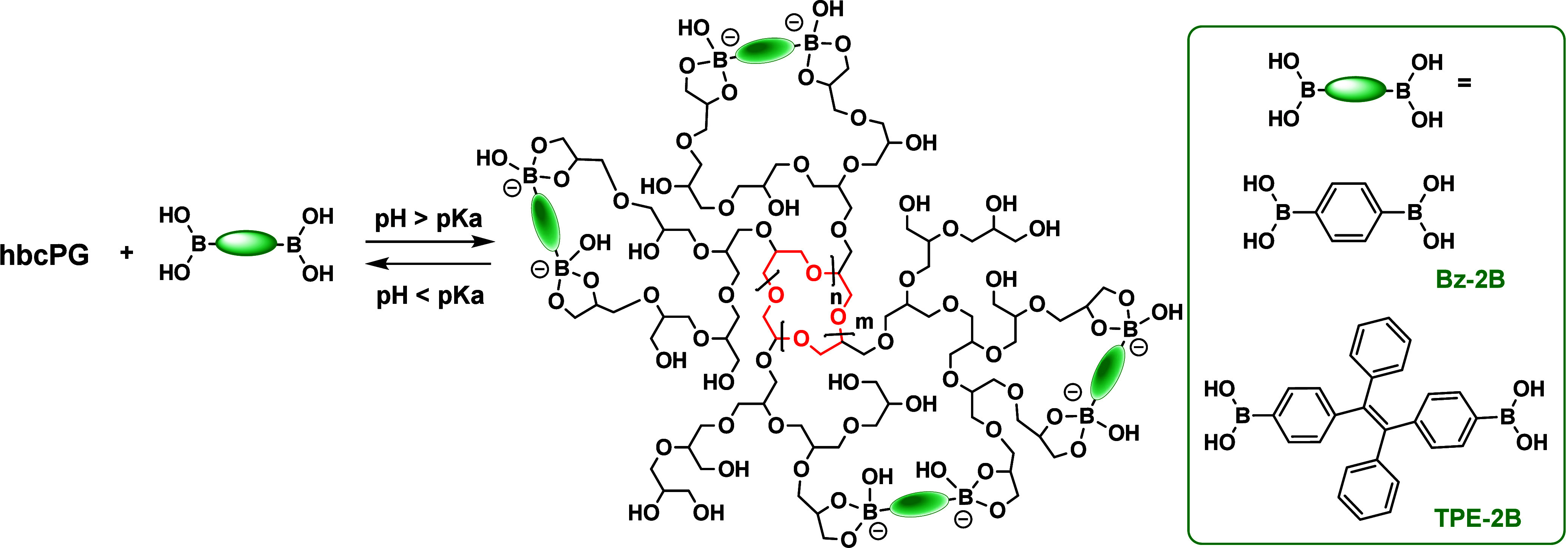
Reversibility of nanogels formed by the intramolecular cross-linking of (h)­bcPG with diboronic acids.

**Figure 4:**
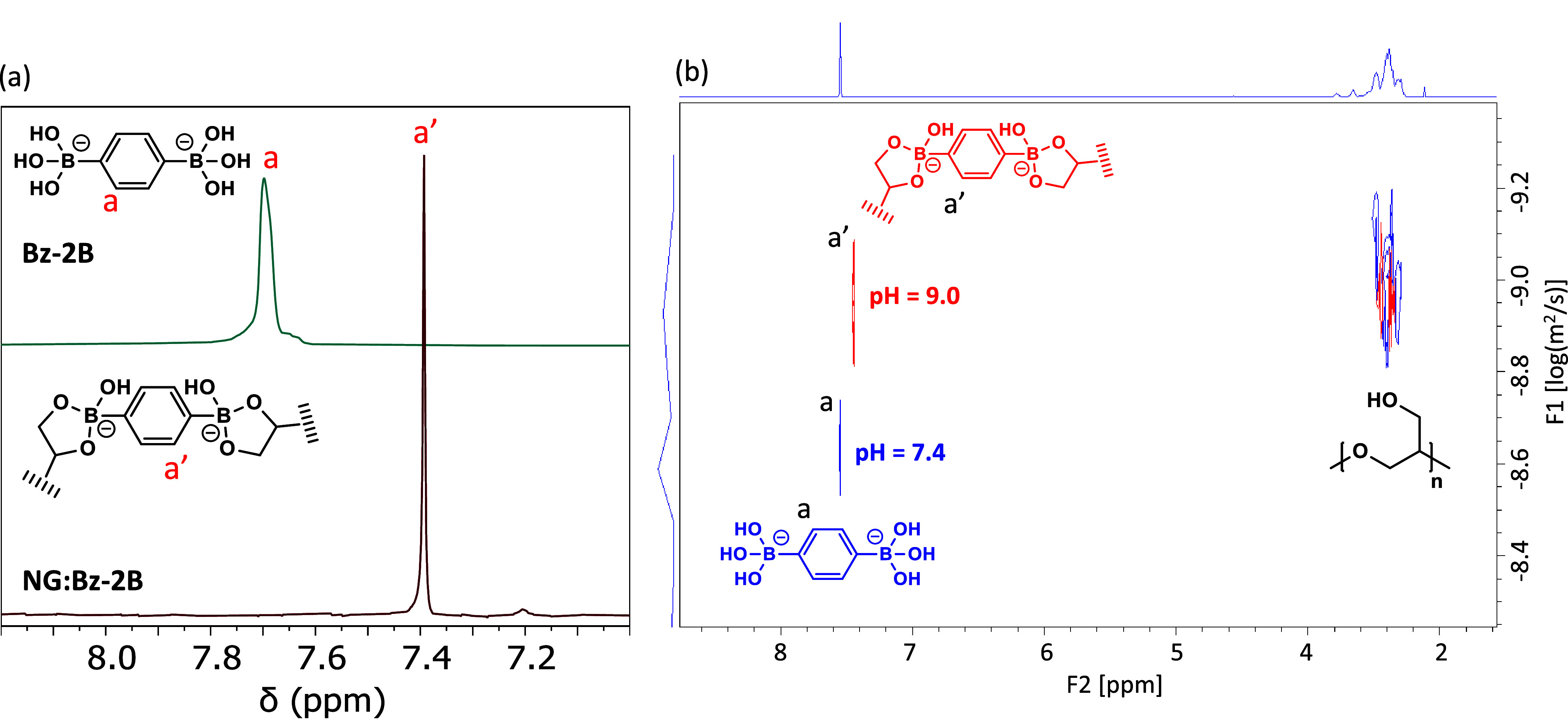
(a) ^1^H NMR of Bz-2B and nanogel NG_10k_:Bz-2B in a carbonate buffer of pH = 9 (5 vol % of DMSO-*d*_6_). Spectra were normalized to DMSO signal intensity. (b) DOSY NMR spectra of NG_10k_:Bz-2B ([boronic acid]/[diol] = 0.8) obtained in D_2_O at pH = 7.4 and 9.

The reversibility of the reaction was further demonstrated by a DOSY NMR experiment (Figure 4b). At pH 9, the ^1^H signal (a′) assigned to the Bz-2B moiety anchored to a boronic ester showed a diffusion comparable to that of the bcPG protons, consistent with covalent attachment. Upon acidification to pH 7.4, the ^1^H signal (a) shifted downfield, and the proton diffusion became faster, indicating dissociation of the boronic ester and the regeneration of the boronic acid.

### Nanostructural Characterization by Small-Angle X-ray Scattering (SAXS)

Figure 5 shows the scattering curves of the bcPG and hbcPG nanogels compared to their respective precursors in buffer at a selected polymer concentration of 2 mg/mL (all polymer concentrations are reported in Figure S5). The scattering patterns of the precursors do not change in buffer relative to plain water (Figure S6), indicating no significant effect of the solvent on the polymer conformation. As the molecular weight of the branched polymer increases, the SAXS profiles evolve from a slope of approximately *Q* ^–1.7^, characteristic of typical polymer-like behavior for low-molecular-weight bcPG,^36^ to slopes closer to *Q* ^–4^ indicative of compact, spherical objects in the case of high-molecular-weight hbcPG. The SAXS profiles of the nanogels show no significant changes upon cross-linking compared to their corresponding precursors, suggesting that nanogel formation preserves the overall size and conformation of the precursor polymers.

**Figure 5:**
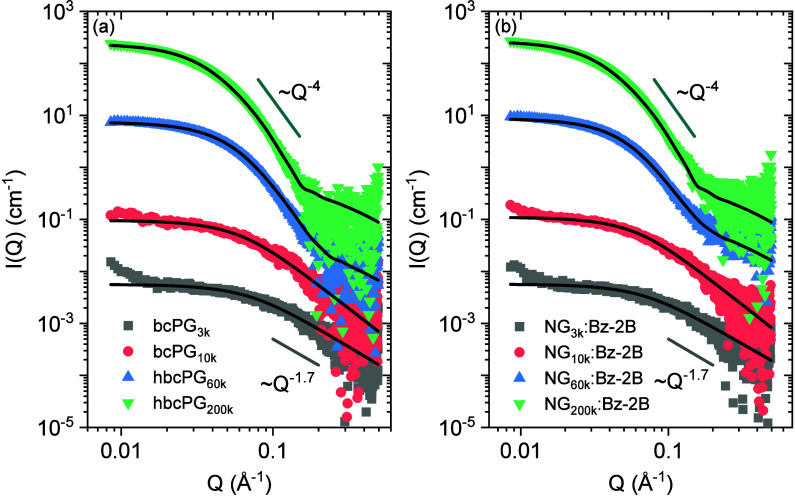
SAXS profiles of bcPG and hbcPG precursors (a) and nanogels NG:Bz-2B (b) in a carbonate buffer at pH 9 and at a polymer concentration of 2 mg/mL. bcPG low molecular weight samples (3 and 10 kDa, entries 1 and 2, Table 1) were fitted with a generalized Debye model. The data for the hbcPG high molecular weight samples (60 and 200 kDa, entries 4 and 5, Table 1) were fitted with a polydisperse fuzzy-sphere model. The solid gray line corresponds to a scaling of *I* ∼ *Q* ^–1.7^, and the green line to ∼*Q* ^–4^ serves as references. SAXS curves of the lowest molecular weight samples are in absolute scale and the rest have been shifted vertically (x10^n^) for better visualization.

In order to obtain more information about the structure and shape of the nanogels, we analyzed the SAXS data over the entire *Q* range using the appropriate form factors. As discussed above, the structure of the samples depends on the molecular weight, and we identified two regimes based on this parameter. While the samples with low molecular weight (3 and 10 kDa) resemble polymer fractals, polymers with larger molecular weights (60 and 200 kDa) attain a more compact, spherical structure. For the former we employed a generalized Debye form factor that describes the scattering from polymers with arbitrary chain statistics characterized by the radius of gyration, *R*_g_, and the fractal dimension, *d*_f_ related to the Flory exponent by *d*_f_ = 1/ν. The fit results, summarized in Table 2, show that the fractal dimension increases with molecular weight, indicating that the structure becomes more compact, in line with expectations derived from the synthesis design. The compaction of the polymeric structure becomes more pronounced at larger molecular weights, where the analysis using a chain form factor is inadequate. Instead, the data are effectively described by a spherical form factor with a diffuse outer surface. This model, referred to as the fuzzy sphere model (see Materials and Methods), is characterized by the radius *R*_p_ and width of the Gaussian interface σ_Rp_ along with a polydispersity that follows a Gaussian distribution with a relative width, σ_PD_. The resulting fit analysis yields very good agreement with the experimental data after the inclusion of a “blob” contribution originating from the individual chain segments of size ξ ∼ 0.5 nm within the nanogel. The important fit parameters are listed in Table 3. The fit results show that the nanogels can be characterized by polydisperse spheres with broad interfaces of the 30–50% width with respect to the average size.

**Table 2:** Results from Form Factor Analysis of Polymer Fractal-like Structures

entry	structure	*M*_w_ (kDa)	*R*_g_ (Å)	*d*_f_
1	bcPG_3k_	4.4 ± 0.5	20.1 ± 2.2	1.8 ± 0.2
1	NG_3k_:Bz-2B	4.9 ± 0.5	20.3 ± 2.0	1.7 ± 0.2
2	bcPG_10k_	7.5 ± 1.5	25.6 ± 1.5	2.6 ± 0.2
2	NG_10k_:Bz-2B	9.6 ± 1.5	25.7 ± 1.5	2.3 ± 0.2

**Table 3:** Results from Form Factor Analysis to a Fuzzy Sphere Model

entry	structure	*M*_w_ (kDa)	*R*_p_ (Å)	σ_Rp_ (Å)	σ_PD_
4	hbcPG_60k_	57 ± 5	29 ± 2	12 ± 2	0.5 ± 0.1
4	NG_60k_:Bz-2B	77 ± 5	30 ± 3	13 ± 2	0.5 ± 0.1
5	hbcPG_200k_	181 ± 10	42 ± 3	18 ± 2	0.6 ± 0.1
5	NG_200k_:Bz-2B	222 ± 10	43 ± 3	17 ± 2	0.6 ± 0.1

The chain conformation was further investigated using a scaling analysis of the radius of gyration as a function of molecular weight based on a model-independent Guinier analysis. The data shown in Figure 6 show a scaling behavior of *R*_*g*_ ∼ *M*_w_^1/3^ which is distinctly different from the scaling expected from a random, Gaussian chain *R*_*g*_ ∼ *M*_w_^1/2^ (lines shown as reference in Figure 6). This is consistent with gradually more compact structures with increasing molecular weight. Interestingly, the precursor (in water and buffer) shows essentially the same scaling behavior as the cross-linked nanogel. The compact structure is furthermore reflected in the ratio of the radius of gyration and hydrodynamic radius obtained by dynamic light scattering *R*_g_/*R*_h_ ∼ 0.78 (Table 1) which is within the characteristic values for globular structures.^50^ It is also worth noticing that cross-linking leads to an increase of the *M*_w_ as expected, but to a very small increase in *R*_g_ indicating that the boronic cross-linker inserts within the nanogel entities.

**Figure 6:**
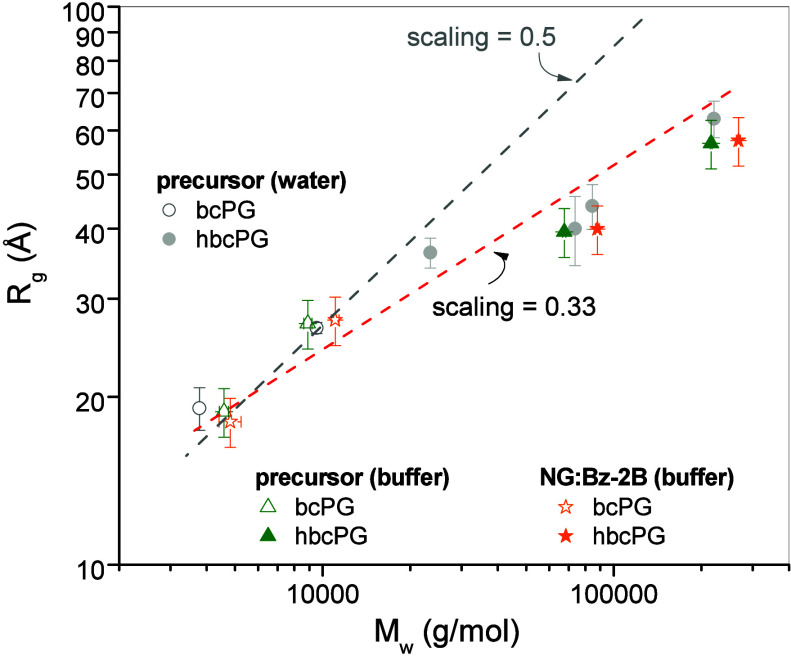
The log–log plot of *R*_g_ vs *M*_w_ obtained by the Guinier analysis of the SAXS data for bcPG and hbcPG in water and in buffer, and their nanogels NG:Bz-2B. Dash lines are data fitting obtained by fixing the scaling exponent to a value of 0.33. For comparison, the line with a scaling exponent of 0.5 is also shown.

However, we note a discrepancy between the fractal dimensions obtained from the Debye model fits for the low molecular weight samples (*d*_f_ ≈ 1.7–2.3, closer to expectations for polymers in good solvent, ν ≈ 0.6) and the scaling exponent from the Guinier analysis (*R*_g_ ∼ *M*_w_^0.3^). This difference may arise because the available data at low *M*_w_ values are too limited to capture the correct scaling regime. Alternatively, it may reflect the branched growth that starts from the same bcPG_3k_ precursor, which inherently leads to a more spherical architecture with increasing molecular weight, ultimately resulting in an effective scaling exponent closer to 1/3.

Importantly, the very small increase in *R*_g_ upon cross-linking, together with the concentration invariance of *R*_g_ and the absence of concentration-dependent changes in size and low-*Q* scattering upturns (see Figure S5), provides strong evidence that the diboronic acid-mediated cross-linking proceeds intramolecularly. Complementary static light scattering (SLS) measurements (Figure S7), which offer enhanced sensitivity in the lowest-*Q* regime, reveal only a weak low-*Q* excess that is already present in the precursor solutions and is slightly enhanced after cross-linking, consistent with weak interparticle interactions rather than the formation of large aggregates. The preservation of the overall dimensions and the architecture-dependent form factors indicates that nanogel formation proceeds predominantly intramolecularly with no significant interparticle association or network formation.

A complementary SAXS study performed on conventional hyperbranched polyglycerols (hbPG) with a three-arm core, at both low (12 kg mol^–1^) and high (50 kg mol^–1^) molecular weights, revealed a response to cross-linking fully consistent with that observed for the (h)­bcPG architectures with a cyclic core (Table S5, Figure S10). In all cases, cross-linking leads to only marginal changes in the radius of gyration, within experimental uncertainty (e.g., *R*_g_ = 47.8 Å for hbPG_50k_ vs 48.1 Å for the corresponding nanogel), accompanied by a modest increase in molar mass (*M*_w_/*M*_w0_ ≈ 1.1–1.2). This quantitative behavior is observed at both molecular weights investigated and is consistent with the incorporation of the cross-linkers within individual macromolecules. These results support the conclusion that nanogel formation proceeds intramolecularly and that the structural response to cross-linking is, as expected, largely insensitive to the underlying core topology, being instead governed by global architectural constraints.

### Fluorescent Nanogels

The incorporation of AIE-active diboronic acids provides a sensitive probe to investigate the internal structuring of the nanogels formed by the intramolecular cross-linking of (h)­bcPGs. In particular, TPE-2B serves a dual role as a dynamic covalent cross-linker and a fluorescent probe whose emission intensity and spectral position are highly sensitive to local confinement and microenvironment polarity. The RIR enhancement caused by the restricted rotation of TPE-2B’s phenyl groups upon chemical cross-linking enables the identification of accessible sites within the (h)­bcPG networks where their motion is constrained. For this purpose, fluorescent nanogels were obtained by reacting TPE-2B with (h)­bcPG of different molecular weights. SAXS data of nanogels synthesized from bcPG_3k_ and hbcPG_200k_ at a polymer concentration of 3 mg/mL (Figure S8 and Tables S2 and S3) showed that they retain the overall size and conformation of the precursors, indicating that intramolecular cross-linking occurs within individual (h)­bcPG structures without inducing aggregation.

The behavior of TPE-2B in aqueous solution was consistent with previously reported data.^27,42^ TPE-2B (50 μM) exhibited intense light emission at pH 7 under UV lamp irradiation. When the pH was increased to 10, the fluorescence was quenched (Figure 7a). Finally, upon the addition of hbcPG to the TPE-2B solution at pH 10, the light emission increased again. The emission intensity increased with the amount of hbcPG, as shown in Figure 7b for hbcPG_200k_. Furthermore, the emission intensity and emission wavelength were found to also depend on the molecular weight of (h)­bcPG structures (Figure 7c) at identical TPE-2B and polymer concentrations, fixed at 50 μM and 3 mg/mL, respectively.

**Figure 7:**
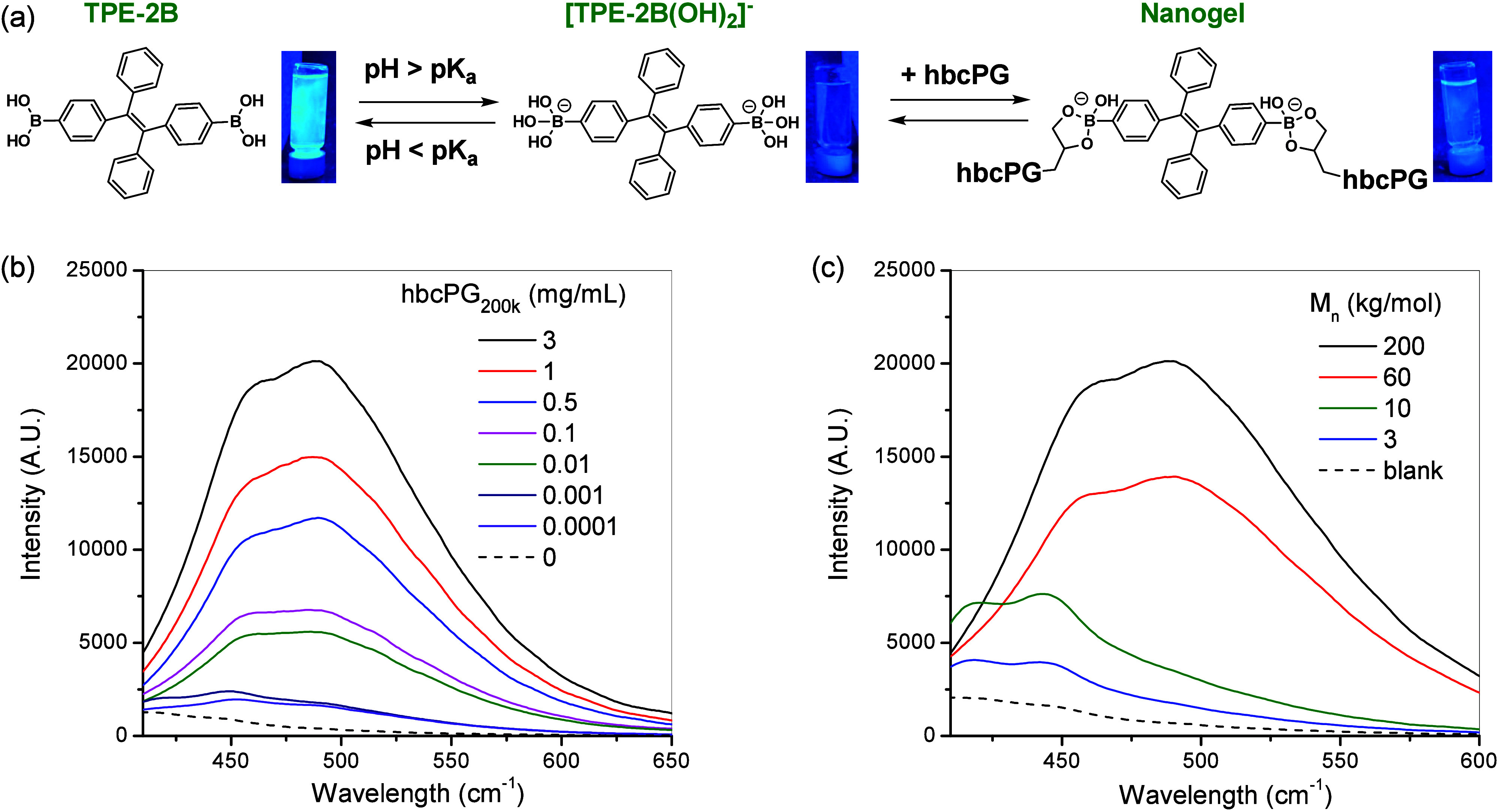
(a) Schematic representation of the acid-basic equilibria of TPE-2B and the nanogel accompanied by the images of TPE-2B (50 μM) solutions under UV light at pH 7 (TPE-2B), at pH 10 (TPE-2B­(OH)_2_^–^), and in the presence of hbcPG_200k_ at pH 10 (Nanogel). (b) Fluorescence spectra (λ_exc_ = 365 nm^–1^) of nanogels obtained by reaction of hbcPG_200k_ and 50 μM of TPE-2B at different polymer concentrations in a buffer of pH 10. (c) Fluorescence spectra for different molecular weight (h)­bcPG structures (3 mg/mL) obtained at [TPE-2B] = 50 μM and pH 10.

The TPE moiety is a well-known AIE fluorogen whose fluorescence is triggered through the RIR effect.^27,42,51^ At pH values above its p*K*_a_ (around 8.6–9), boronate anions are formed (TPE-2B­(OH)_2_^–^, Figure 7), rendering the molecule fully water-soluble. Under these conditions, the fluorogen remains nonemissive. However, below its p*K*_a_, the molecule becomes poorly soluble and aggregates, promoting AIE. Upon the addition of (h)­bcPG a different effect is observed. In this case, the boronate anions formed at high pH react with the diol groups of the polymer to yield dynamic covalent boronic esters. This covalent interaction restricts the intramolecular rotation of the phenyl groups of TPE-2B units, leading to enhanced fluorescence. Given that TPE-2B is a bifunctional molecule and the (h)­bcPG structures contain multiple reactive diol groups, the number and type of binding between the fluorogen and the polymer can vary. TPE-2B may exist in dynamic equilibrium among unbound species and species bound through one or both of its boronic acidic functions, depending on the local diol availability within the (h)­bcPG structure.

Figure 7b clearly shows that the emission intensity of TPE-2B increases with increasing hbcPG_200k_ concentration, indicating that a progressively larger fraction of TPE-2B becomes covalently attached to the diol groups of hbcPG_200k_. At the maximum polymer concentration (3 mg/mL), the (h)­bcPG structures exhibit lower emission intensity and blue-shift emission maxima with decreasing molecular weight (Figure 7c). It is important to note that the relative diol content decreases when moving from hbcPG to bcPG architectures, as derived from the structural unit analysis based on inverse-gated ^13^C NMR spectroscopy (Table 1). The lower density of diol functionalities in bcPG therefore provides fewer binding sites for diboronic acid cross-linkers, which reduces the anchoring probability of the TPE-2B molecules.

The hypsochromic shift observed for bcPG compared to hbcPG structures can be partially attributed to differences in the local environment of TPE-2B, consistent with previous observations for TPE analogues.^52^ These results suggest that hbcPG structures are able to host TPE-2B molecules within their interior, which present lower polarity than the polymer periphery, in agreement with our previous study on the interaction of hydrophobic probes with branched and hyperbranched polyglycerol.^38^ This effect is similar for hbcPG_60k_ and hbcPG_200k_, as well as for bcPG_3k_ and bcPG_10k_, suggesting that within each polymer type, the fluorophore experiences comparable microenvironments. That is, hbcPG structures accommodate TPE-2B molecules within interbranch hydrophobic domains, whereas bcPG structures host them at the polymer periphery in a highly polar environment.

By exploring a wider range of polymer concentrations [PG] for the four molecular weights under study, the results exhibit clear differences (Figure 8a). In the analysis, it is important to note that each data point was obtained through independent experiments involving the synthesis of fresh nanogels at the specified concentrations. The figure shows the fluorescence intensity ratio (*I*/*I*_0_) of the maximum at 444 nm for bcPG_3k_ and bcPG_10k_, and at 490 nm for hbcPG_60k_ and hbcPG_200k_, and free TPE-2B in solution, plotted as a function of [PG]. Figures 8b and S9 present alternative representations of these data as a function of the molar equivalent ratio of boronic acid to diol functionalities [boronic acid]/[diol] and the molar ratio of TPE-2B to (h)­bcPG ([TPE-2B]/[PG]), respectively. In general, the I/I_0_ values are higher for the higher molecular weight (h)­bcPG structures in the entire concentration range, reaching values as high as 50 for hbcPG_200k_ and only 2.4 for bcPG_3k_ at the highest [PG]. Two-step behavior in the fluorescence intensity was revealed in all samples (except the lowest-molecular-weight bcPG_3k_ polymer), as indicated by the arrows marking the onset of the first and second steps. The first fluorescence increase occurs at relatively low polymer concentrations and is characterized by a moderate enhancement of the emission intensity. The second fluorescence step appears at higher polymer concentrations and is accompanied by a pronounced increase in the emission intensity.

**Figure 8:**
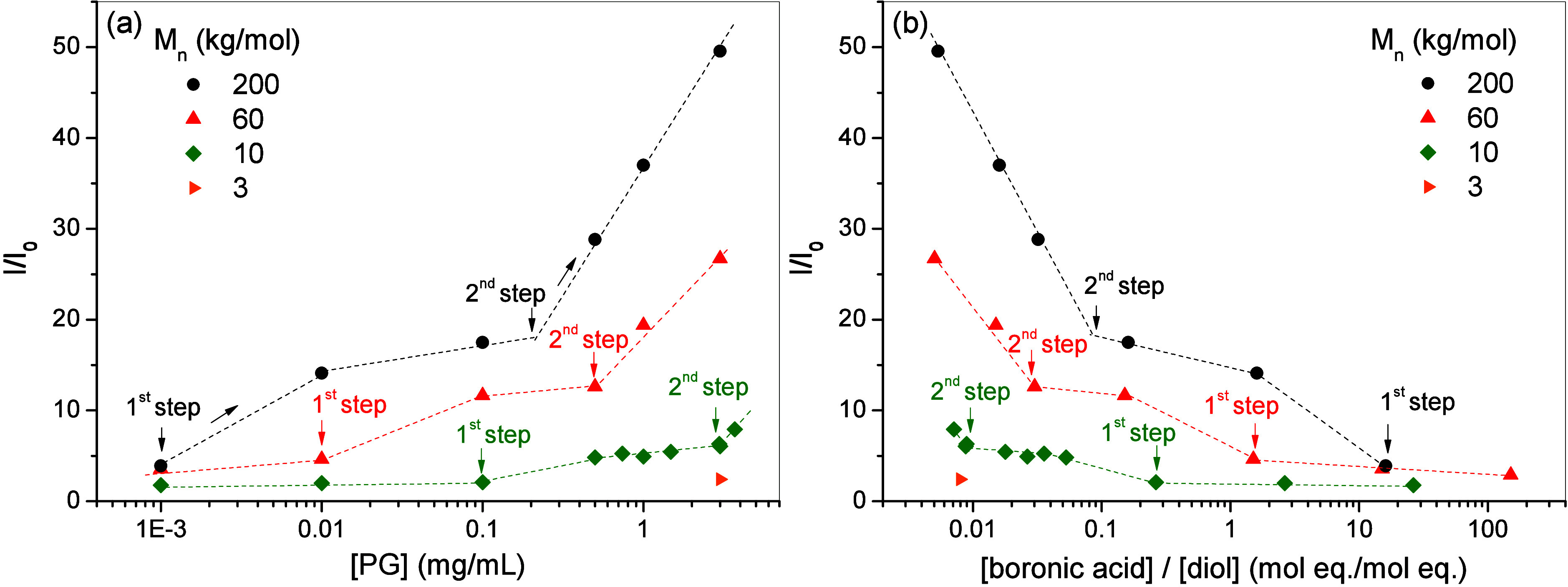
(a) Fluorescence intensity ratio (*I*/*I*_0_) of TPE-2B as a function of the polymer concentration [PG] in nanogels NG:TPE-2B obtained at [TPE-2B] = 50 μM and pH 10. (b) Alternative representation as a function of [boronic acid]/[diol]. Variation of *I*/*I*_0_ upon interaction with (h)­bcPG structures of different molecular weights. *I*/*I*_0_ was determined at 444 nm for bcPG_3k_ and bcPG_10k_ and at 490 nm for hbcPG_60k_ and hbcPG_200k_. *I*_0_ is the fluorescence intensity of the TPE-2B solution at pH 10 in the absence of polymer.

Table 4 reports the characteristic ratios at the onset of the first and second fluorescence regimes, expressed as both [TPE-2B]/[PG] and [boronic acid]/[diol]. These values provide insight into the balance between fluorogen availability and reactive diol sites during the progressive anchoring of TPE-2B within the polymer architectures. At the onset of the first fluorescence regime, [boronic acid]/[diol] values larger than unity are observed for high-molecular-weight hbcPG samples, indicating that boronic acid functionalities are present in excess with respect to available diol groups. Under these conditions, competition among TPE-2B molecules for diol sites is favored, leading predominantly to single-point attachment and limited confinement of the fluorogen, which manifests as a modest increase in fluorescence intensity. In contrast, for lower-molecular-weight samples, [boronic acid]/[diol] values already fall below unity, reflecting a relative excess of diol functionalities and a reduced degree of competition for binding sites. Despite this favorable stoichiometry, the fluorescence intensity remains low, indicating that efficient confinement is not achieved, most likely due to the limited branch size and the less compact structure of these polymers (as determined by SAXS).

**Table 4:** Number of TPE-2B Molecules Per Polymer at the Onset of the 1st and 2nd Step of *I*/*I*_0_ Increment

	[TPE-2B]/[PG] (mol/mol)		[boronic acid]/[diol] (mol equiv/mol equiv)	
*M*_n_ of (h)bcPG in NG:TPE-2B samples	onset 1st step	onset 2nd step	onset 1st step	onset 2nd step
200k	10300	53	16	0.08
60k	320	6	1.5	0.03
10k	5	0.2	0.3	0.009

At the onset of the second fluorescence regime, [boronic acid]/[diol] ratios are consistently below unity for all polymer architectures, indicating that diol groups are present in excess with respect to boronic acid functionalities. This shift favors more effective anchoring of TPE-2B molecules, including multivalent interactions and enhanced confinement within the polymer architecture, resulting in the pronounced fluorescence enhancement characteristic of the second regime. The systematic dependence of these ratios on the molecular weight highlights the role of polymer architecture in controlling fluorogen confinement. Higher-molecular-weight hbcPG samples, which contain a larger number of diol groups per macromolecule and a more confined internal structure, reach the regime of effective fluorogen confinement at lower polymer concentrations, whereas lower-molecular-weight architectures require higher concentrations to achieve comparable restriction of intramolecular motion (see Figure 8a).

### Physical Entrapment of Rhodamine-B in the Nanogels

The RhB release experiments were designed as a structure–property probe to assess how dynamic intramolecular cross-linking influences molecular transport within ultrasmall nanogels. Rather than aiming to establish a comprehensive drug-release model, this study focuses on identifying characteristic time scales and release regimes associated with free diffusion and network-mediated retention as a function of cross-linking density and pH. hbcPG (60k and 200k) were selected for this study based on the previous results with fluorescent nanogels, which indicated that the diboronic molecule is effectively internalized within the polymer structure. Bz-2B diboronic acid was chosen as the cross-linking agent. Three nanogels were prepared from hbcPG_60k_ in the presence of RhB using different amounts of Bz-2B (Table 5), with [boronic acid]/[diol] molar equivalent ratios corresponding to the range between the first and second step of concentrations observed for analogous fluorescent nanogels (entries C, D, E) and one nanogel from hbcPG_200k_ (entry G). The accumulated fraction of RhB released from these nanogels (Figure 9) was monitored by UV–vis spectroscopy using the experimental setup illustrated in Figure 1.

**Table 5:** Molar Concentration of Species Used in the RhB Release Assays^a^

entry	materials	pH	[boronic acid]/[diol] (mol equiv/mol equiv)	*a* (%)	1/*b* (h)	*d*	*c* (%)	1/*p* (h)	*t*_0_ (h)
A		9	0/0	100​^a^	55.6	1.22			
B	hbcPG_60k_	9	0/1	95​	64.1	1.22^a^			
C	NG_60k_:Bz-2B	9	0.4/1	53​	33.3^a^	1.22^a^	36​^a^	18.5	138
D	NG_60k_:Bz-2B	9	0.5/1	56​	33.3^a^	1.22^a^	36​^a^	21.7	159
E	NG_60k_:Bz-2B	9	0.8/1	54​	33.3^a^	1.22^a^	36​^a^	30.3	166
F	NG_60k_:Bz-2B	7.4	0.5/1	57​	33.3^a^	1.22^a^	36​^a^	22.2	106
G	NG_200k_:Bz-2B	9	0.5/1	52​	33.3^a^	1.22^a^	36​^a^	30.6	184

a Fitting parameters are given in eq 7.

b Fixed parameters during fitting.

**Figure 9:**
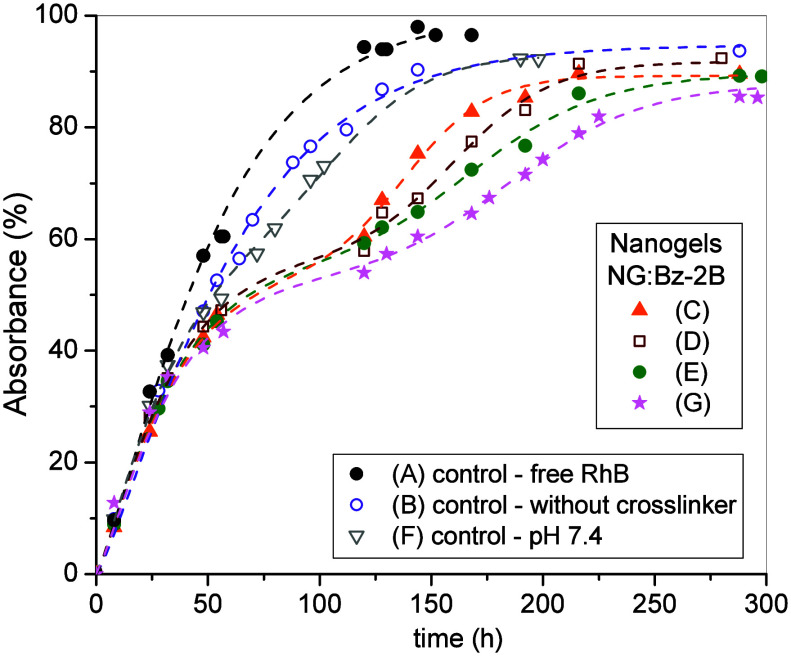
Release of RhB from NG:Bz-2B in a buffer at pH 9 (entries C, D, E, and G, Table 5). Control experiments (A, B, and F). Dashed lines are the fits obtained using eq 7.

As control experiments, the permeation of free RhB through the dialysis membrane was monitored (entry A). In addition, the release of RhB from a physical mixture with hbcPG_60k_ was evaluated as a non-cross-linked reference system (entry B). Finally, RhB encapsulated in a nanogel synthesized at a physiological pH (7.4, Entry F) was investigated. At this pH, which lies below the p*K*_a_ of the boronic acid moieties, the formation of boronic esters is not favored, resulting in a reduced degree of network cross-linking. The release kinetics were analyzed using a purely phenomenological, two-step approach, in which the experimental data were fitted to the sum of an exponential term and a sigmoidal term (eq 7).

7 $$A\left(\%\right)=a\left(1-e^{-{\left(bt\right)}^{d}}\right)+\frac{c}{1+e^{-p\left(t-t_{0}\right)}}$$

*A*(%) is the absorbance (%), *t* is the experimental time, *t*_0_ is the center of the sigmoid, *c* is the amplitude of the sigmoid, and *a*, *b*, *d*, and *p* are coefficients. This choice reflects the experimental observation of two distinct release regimes. This formulation was not intended to represent a mechanistic controlled-release model but rather to provide an empirical description of the characteristic time scales associated with the different release processes. The first exponential term accounts for the diffusion-controlled permeation of free RhB through the dialysis membrane, while the sigmoidal term describes the delayed release of RhB physically entrapped within the nanogel network. Parameters associated with free diffusion were fixed based on control experiments, whereas time-dependent parameters related to the sigmoidal term were allowed to vary to capture the effect of cross-linking density.

The results show that the release of RhB from the nanogels prepared at pH 9 is the slowest of the series compared to the control experiments. Notably, the initial release regime (first step) closely follows the diffusion behavior of free RhB through the dialysis membrane, indicating that this early stage is not affected by the nanogel structure. In contrast, the second release step is systematically shifted toward longer times, in line with the higher boronic acid content in these samples. This result is consistent with an increased extent of boronic ester formation at elevated pH, leading to a higher cross-linking density within the nanogel network and, consequently, to more restricted dye diffusion at longer times. By comparison, at pH 7.4 the release profile of RhB closely resembles that observed for the non-cross-linked polymer system, consistent with the limited formation of boronic ester cross-links under these conditions.

The fitting results are summarized in Table 5. The exponential term accurately describes the permeation of free RhB, with an increase in the parameter 1/*b* (h) observed in the presence of hbcPG (without cross-linking, entry B), reflecting a longer characteristic release time compared to free RhB (entry A). In addition, the presence of hbcPG (entry B) leads to the retention of approximately 5% of RhB within the polymer branches, as evidenced by a plateau that reaches about 95% rather than 100%. For the nanogels, the fitting results confirm a two-step release process. Approximately 50% of the total RhB is released during the initial step, with the remainder released in the second step. The plateau reaches around 90%, indicating that roughly 10% of the RhB remains entrapped within the nanogel structure. The release behavior exhibits a clear dependence on the amount of Bz-2B. This dependence is captured by the parameter *t*_0_ (h), which increases with an increasing Bz-2B content, indicating a shift of the second release step toward longer times. Consistently, the parameter 1/*p* (h) reflects a progressively slower release process as the cross-linking density increases. By increasing the molecular weight of hbcPG from 60k to 200k while maintaining a constant [boronic acid]/[diol] molar equivalent ratio (sample D vs sample G), a further delay of the second release step was observed, indicating that increased polymer size and internal confinement enhance RhB retention within the nanogel and slow its diffusion-controlled release. Finally, a comparative RhB release experiment using nanogels prepared from hbcPG_60k_ and hbPG_50k_ precursors exhibited very similar release profiles for both systems (Figure S11), indicating diffusion and network characteristics. This result further supports the conclusion that the topology of the core (cyclic vs three-arm) does not play a dominant role in this property and that guest release is governed primarily by the overall network structure rather than by the presence of a cyclic core.

## Conclusions

This study demonstrates that intramolecular cross-linking of (hyper)­branched cyclic polyglycerols with diboronic acids yields ultrasmall nanogels whose structure and function are governed by molecular weight and cross-linking density. By combining SAXS, fluorescence probing, and controlled release experiments, we establish a coherent structure–property framework that supports nanogel formation via predominantly intramolecular cross-linking, with no significant contribution from aggregation or interparticle network formation, thereby advancing the rational design of dynamic covalent polymer nanostructures. In particular, we have obtained ultrasmall nanogels (*R*_g_ < 6 nm) from branched and hyperbranched cyclic polyglycerols with molecular weights ranging from 3k to 200 kg/mol. Intramolecular cross-linking of polyglycerol diol groups with diboronic acids (Bz-2B and TPE-2B) under basic conditions led to nanogel formation. High-molecular-weight hyperbranched cyclic polyglycerols adopted compact, globular conformations with nanoparticle-like characteristics, as evidenced by SAXS form factor analysis and scaling of the radius of gyration. In contrast, low-molecular-weight branched cyclic polymers displayed more open, fractal-like conformations consistent with polymeric chains in good solvent conditions.

The photophysical response of the aggregation-induced emission-active diboronic acid TPE-2B provided insight into nanogel structuring. Low-molecular-weight branched cyclic polyglycerols predominantly accommodate TPE-2B at the periphery, whereas high-molecular-weight analogues confine the cross-linker within their interior, leading to enhanced fluorescence emission due to restricted intramolecular rotation of TPE-2B phenyl groups. Furthermore, encapsulation and release experiments using rhodamine-B demonstrate that dynamic intramolecular cross-linking influences molecular retention within the nanogels, with release behavior governed by cross-linking density and molecular weight. At physiological pH, limited boronic ester formation results in reduced encapsulation efficiency, defining clear boundaries for effective nanogel formation under these conditions. This limitation is not specific to Bz-2B but reflects the general behavior of boronic ester equilibria below the p*K*_a_ of the boronic acid, indicating that future improvements will rely on stabilizing boronic ester formation through molecular design and environmental control.

## Supplementary Material



## Notes

Supporting figures containing MALDI-ToF MS, inverse-gated ^13^C NMR, ^1^H NMR, SAXS, and fluorescence; supporting tables containing nanogel stoichiometry and relevant experimental data of hbPG bearing three-arm core (PDF)
