# Supplementary material for: Ultrasmall Nanogels from Intramolecular Cross-Linking of (Hyper)Branched Cyclic Polyglycerols with Diboronic Acids
Source: Macromolecules. 2026 Mar 30;59(7):3975–88. doi: 10.1021/acs.macromol.5c02780 (PMC13086057; doi:10.1021/acs.macromol.5c02780)
Supplement: Supplementary file 1 [file ma5c02780_si_001.pdf]

Ultrasmall Nanogels from Intramolecular Crosslinking of (Hyper)Branched Cyclic Polyglycerols with Diboronic Acids

Eric Gómez Urreizti,[a,b,c] Paula Malo de Molina,*[b,d] Carlo Andrea Pagnacco,[a,b,c] Jose A. Pomposo,[b,c,d] Reidar Lund,[a,e,f] Fabienne Barroso-Bujans*[a,b,c,d]

[a]Donostia International Physics Center (DIPC), Paseo Manuel Lardizábal 4, 20018 Donostia–San Sebastián, Spain

[b]Centro de Física de Materiales (CSIC-UPV/EHU)-Materials Physics Center MPC, Paseo Manuel Lardizábal 5, 20018 Donostia–San Sebastián, Spain

[c]PMAS, Faculty of Chemistry, University of the Basque Country (UPV/EHU), Paseo Manuel Lardizábal 3, 20018 Donostia–San Sebastián, Spain

[d]IKERBASQUE-Basque Foundation for Science, Plaza Euskadi 5, 48009, Bilbao, Spain

[e]Department of Chemistry, University of Oslo, Postboks 1033, Blindern, Oslo, 0315, Norway

[f]Hylleraas Centre for Quantum Molecular Sciences, University of Oslo, Postboks 1033, Blindern, Oslo, 0315, Norway

Table of Contents

1. MALDI-ToF mass spectrometry 2
2. Inverse-gated ^{13}C NMR 2
3. Stoichiometry for nanogel formation 3
4. Supporting ^{1}H NMR figures 4
5. Supporting SAXS figures 4
6. Fluorescence spectroscopy 8
7. Nanogel derived from a three-arm-core hbPG 8

1. MALDI-ToF mass spectrometry

MALDI-ToF mass spectra of bcPG samples (Entries 1 and 2 in Table 1) show peak distributions consistent with M_{obs} (Da) = $nM_{Gly} + M_{Li}$, confirming the cyclic nature of the polymers (C_n), where n is the number of glycidol units, M_{Gly} = 74.08 g mol^{-1}, and M_{Li} corresponds to the lithium cationizing agent (**Figure S1**). For higher-molecular-weight hbcPG samples, the cyclic core is expected to be retained by design across the series, given that these polymers are synthesized from the entry-1 bcPG precursor.

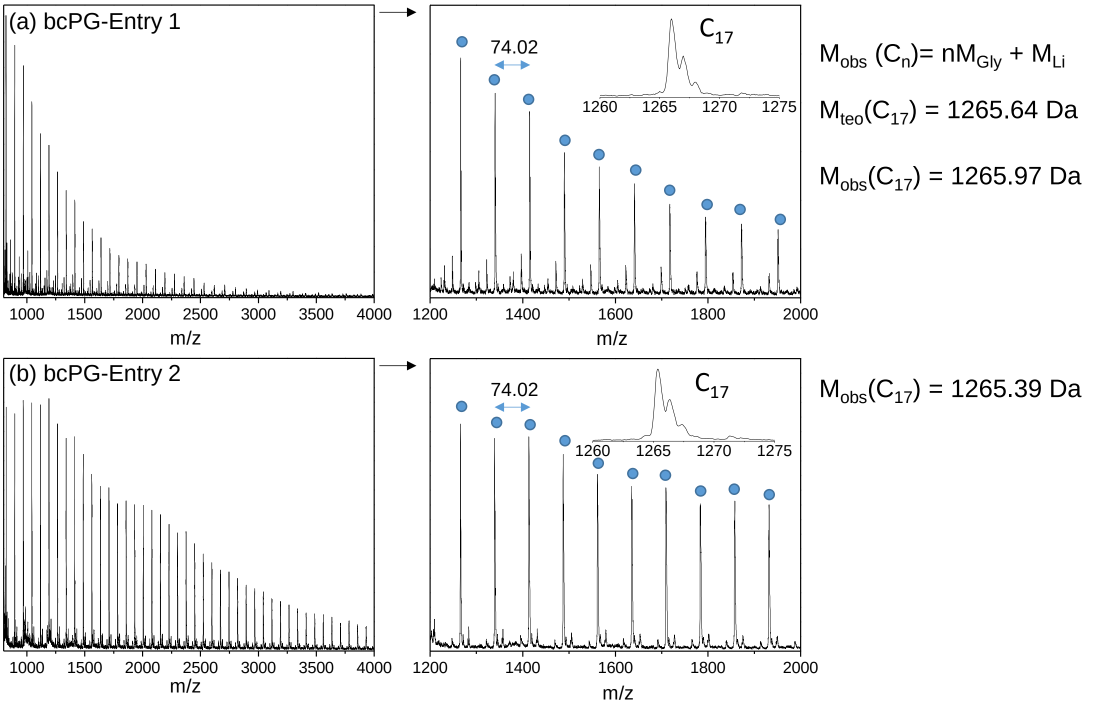

Figure S1. MALDI-ToF MS spectra of bcPG samples (Entries 1 and 2 in Table 1). Insets show expanded regions of the spectra. Lithium trifluoroacetate (LiTFA) was used as a cation donor and 4-hydroxycinnamic acid (CHCA) was used as a matrix as in previous experiments.[1]

2. Inverse-gated ^{13}C NMR

The relative abundance of terminal (T), dendritic (D), and linear units in the polyglycerol architectures was determined from inverse-gated ^{13}C NMR spectra to obtain quantitative signal intensities. Due to the unsymmetrical nature of glycidol, two types of linear units were distinguished, namely $L_{1,3}$ and $L_{1,4}$ linear units, and all resonances were assigned according to established literature procedures.[2,3] The relative fractions of T_1, T_2, D, $L_{1,3}$, and $L_{1,4}$ were obtained by integrating the corresponding ^{13}C signal regions (using non-overlapping resonances whenever possible) and normalizing the integrals to yield the distribution of structural units.

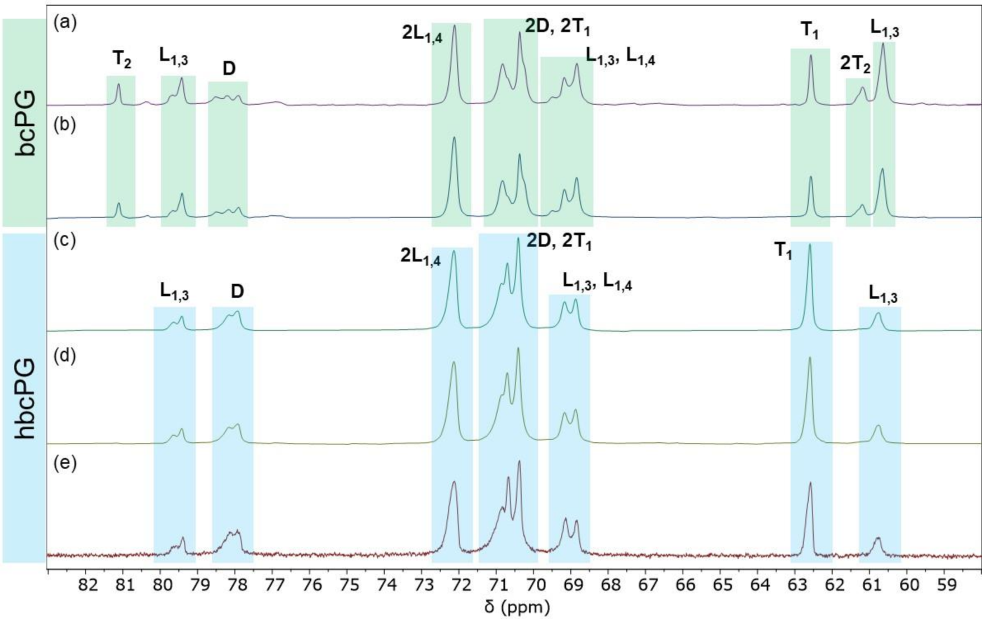

Figure S2. Inverse-gated ^{13}C NMR spectra of bcPG and hbcPG in D_2O corresponding to Entries 1-5 in Table 1. (a) Entry 1, (b) Entry 2, (c) Entry 3, (d) Entry 4, (e) Entry 5. Peak assignments for bcPG and hbcPG were performed according to references 2 and 3, respectively.

3. Stoichiometry for nanogel formation

Table S1. Molar equivalents of boronic acid relative to polymer diol functionalities ([boronic acid] / [diol]) in nanogels obtained by cross-linking (h)bcPG of different molecular weights with Bz-2B (NG:Bz-2B). Polymer concentration was maintained constant.

Entry	Polymer used in nanogels NG:Bz-2B	[boronic acid] / [diol] (mol eq./ mol eq)	
		for SAXS experiments [polymer] = 2 mg/mL	for RhB release [polymer] = 12 μg/mL
1	$bcPG_{3k}$	0.8	-
2	$bcPG_{10k}$	0.9	-
3	$hbcPG_{40k}$	0.5	-
4	$hbcPG_{60k}$	0.5	0.8; 0.5; 0.4
5	$hbcPG_{200k}$	0.5	0.5

4. Supporting ^{1}H NMR figures

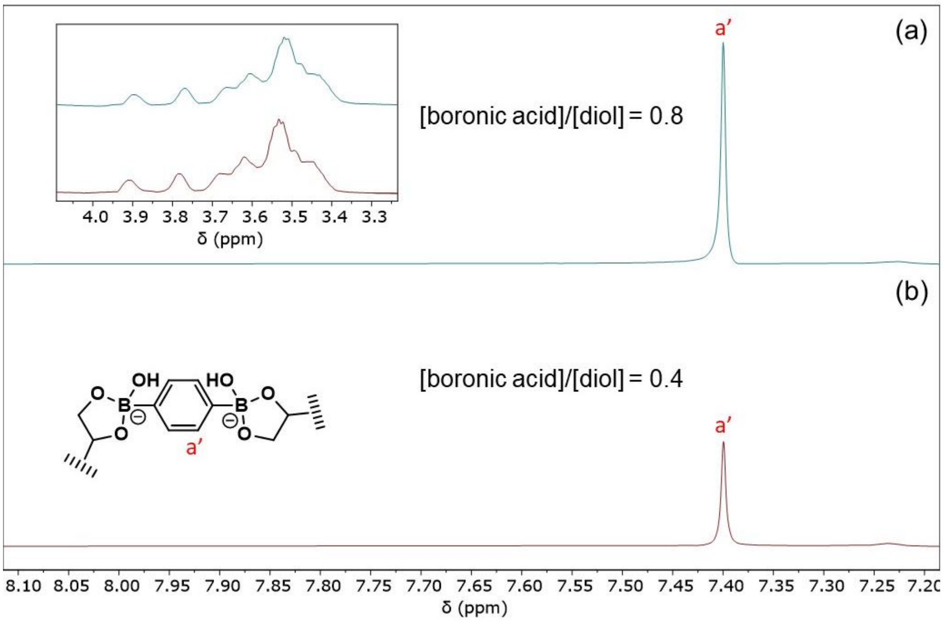

Figure S3. ^{1}H NMR of NG_{60k}:Bz-2B in a carbonate buffer of pH = 9 (5 vol% of DMSO-d_6) showing a decrease in the intensity of the a' signal upon reducing the [boronic acid]/[diol] equivalent ratio from (a) 0.8 to (b) 0.4. Signal intensities were normalized to the CH_2 and CH resonances of the glycidol moieties of the polymer (inset).

5. Supporting SAXS figures

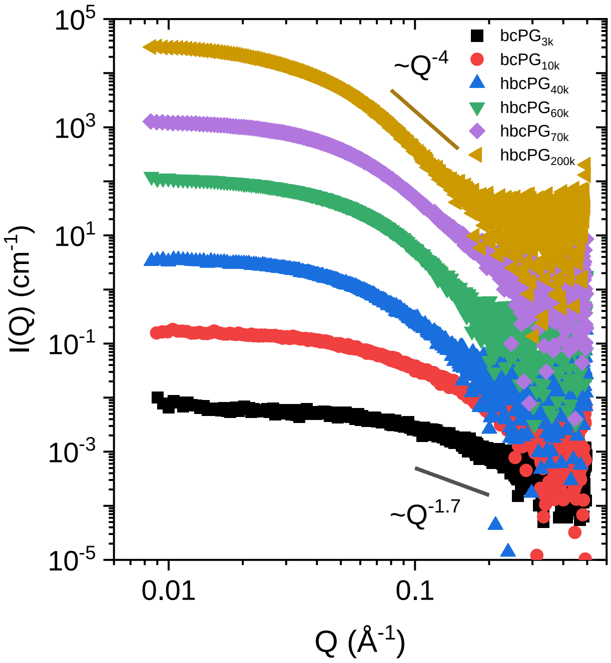

Figure S4. SAXS profiles of bcPG and hbcPG in water at 2 mg/mL (see **Table 1**). The solid gray line corresponds to a scaling of $I \sim Q^{-1.7}$ and the ocre line to $\sim Q^{-4}$ serve as references. SAXS curves of the lowest molecular weight samples are in absolute scale and the rest have been shifted vertically (x10^n) for better visualization.

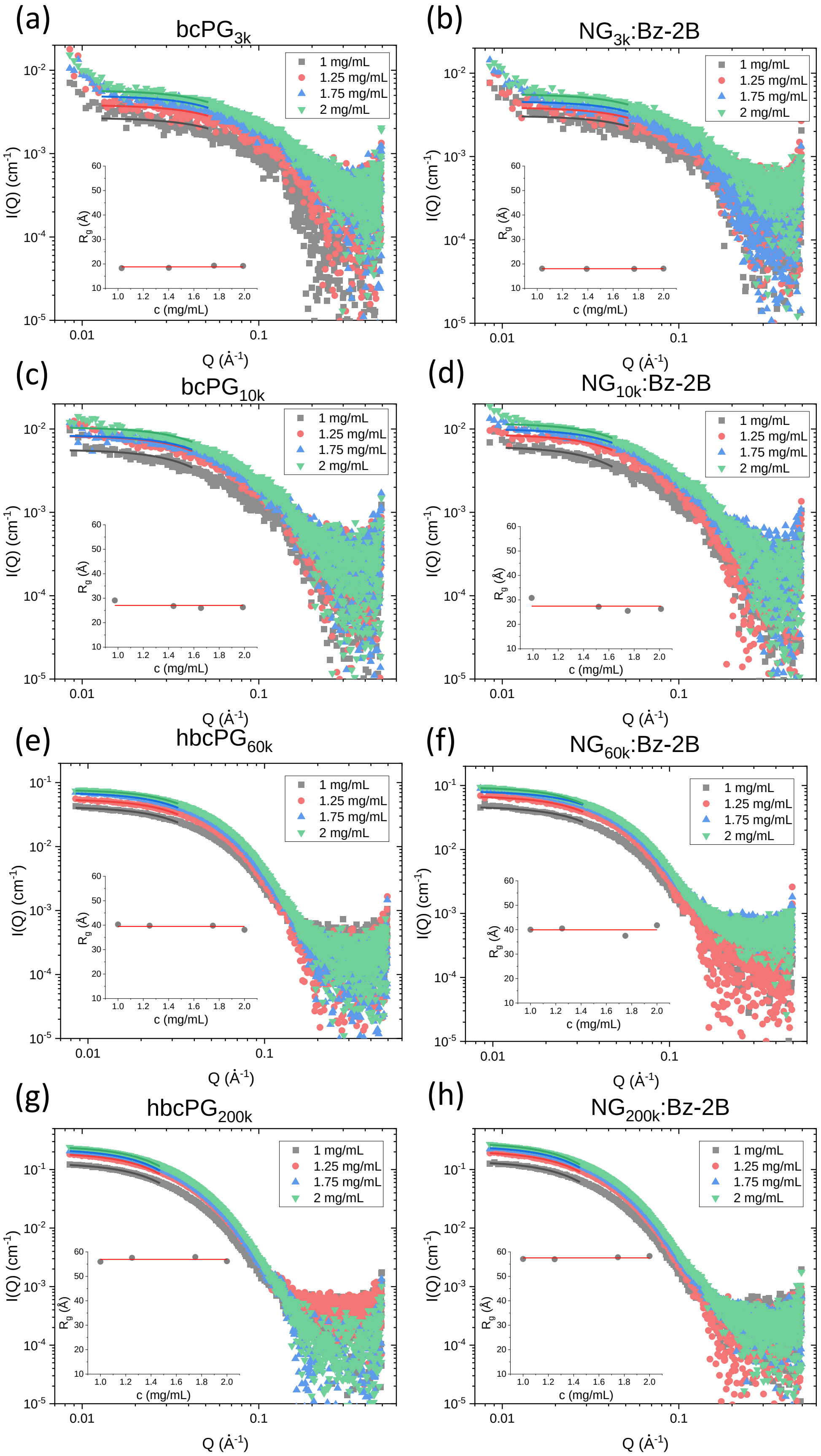

Figure S5. SAXS profiles of bcPG and hbcPG precursors and the corresponding nanogels measured at different concentrations (1–2 mg mL⁻¹) in carbonate buffer of pH 9. Panels

(a,c,e,g) show the bcPG and hbcPG precursors with increasing molecular weight (3k, 10k, 60k, and 200k), while panels (b,d,f,h) show the corresponding nanogels. Symbols correspond to experimental data at different concentrations, as indicated in the legends. Solid lines represent Guinier fits applied in the low-Q regime. Insets display the resulting radius of gyration (R_g) as a function of concentration. The red horizontal line denotes the average R_g value, highlighting the absence of significant concentration dependence.

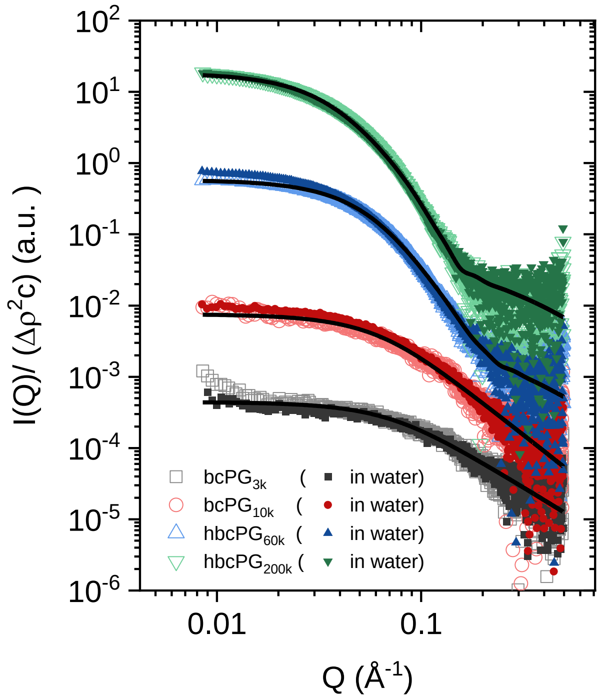

Figure S6. Comparison of SAXS profiles registered in carbonate buffer of pH 9 and water for bcPG and hbcPG (Entries 1, 2, 4 and 5, **Table 1**) at 2 mg/mL. Solid lines correspond to fits: bcPG low molecular weight samples (3 and 10 kDa) were fitted with a generalized Debye model. The data for the hbcPG high molecular weight samples (60 and 200 kDa) were fitted with a polydisperse fuzzy-sphere model. SAXS curves of the lowest molecular weight samples are in absolute scale and the rest have been shifted vertically (x10^n) for better visualization.

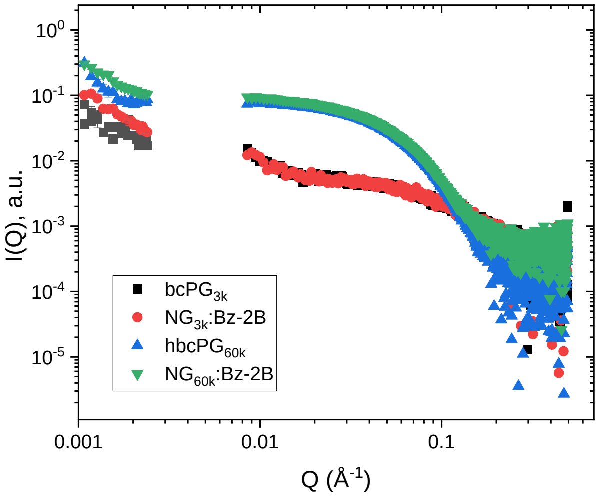

Figure S7. Combined SLS and SAXS intensity profiles, I(Q), as a function of Q for $bcPG_{3k}$, $hbcPG_{60k}$, and the corresponding nanogels (NG_{3k}:Bz-2B and NG_{60k}:Bz-2B). SLS

data in the low-Q region are multiplied by the same constant factor for clarity, while SAXS data are shown on an absolute intensity scale (cm^{-1}).

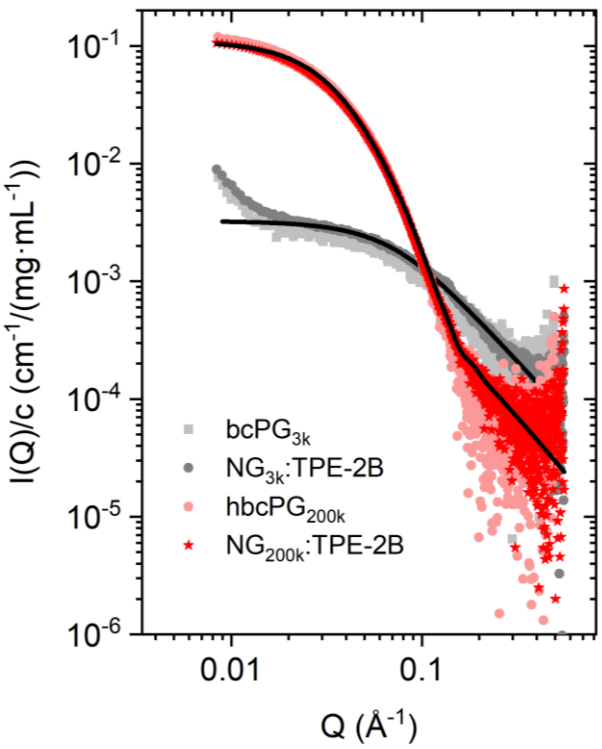

Figure S8. SAXS profiles of fluorescent nanogels NG:TPE-2B at 3 mg/mL in buffer and their corresponding bcPG and hbcPG precursors (Entries 1 and 5, Table 1) at 2 mg/mL in buffer. Solid lines correspond to fits. The data of NG_{3k}:TPE-2B were fitted with a generalized Debye model. The data of NG_{200k}:TPE-2B were fitted with a polydisperse fuzzy-sphere model.

Table S2. Results from form factor analysis of polymer fractal-like structures.

Entry	Structure	M_w (kDa)	R_g (Å)	d_f
1	$bcPG_{3k}$	4.4 ±0.5	20.1±2.2	1.8 ±0.2
1	NG_{3k}:TPE-2B	5.2 ±0.5	19.7±0.4	1.78 ±0.08

Table S3. Results from form factor analysis to a fuzzy-sphere model.

Entry	Structure	M_w (kDa)	R_p (Å)	σ_{Rp} (Å)	σ_{PD}
5	$hbcPG_{200k}$	181 ±10	42 ±3	18 ± 2	0.6 ±0.1
5	NG_{200k}:TPE-2B	183 ±10	40 ±3	18 ± 2	0.6 ±0.1

6. Fluorescence spectroscopy

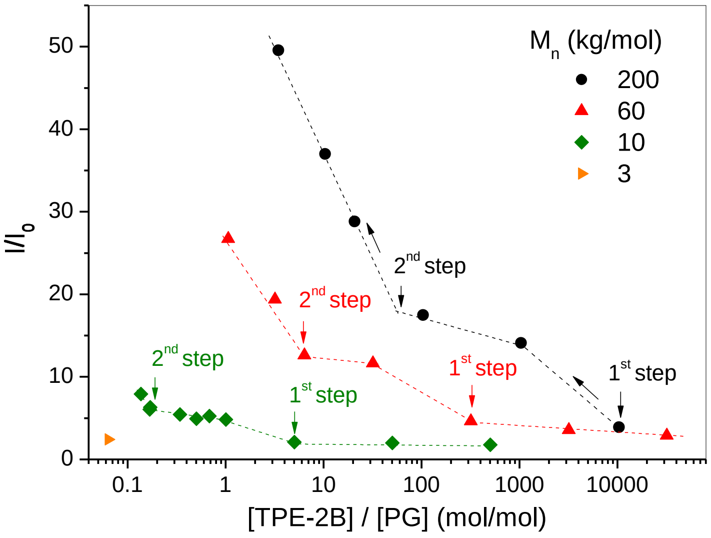

Figure S9. Fluorescence intensity ratio (I/I_0) of TPE-2B as a function of [TPE-2B] / [PG] in nanogels NG:TPE-2B. Complementary data of Figure 8.

7. Nanogels derived from hbPG with a three-arm-core

Synthesis of hyperbranched polyglycerol (hbPG): Two samples of conventional hbPGs bearing a three-arm core were synthesized from 1,1,1-tris(hydroxymethyl)propane (TMP) by ring-opening multibranching polymerization of glycidol in dioxane. In a glove box, TMP (18.8 mg, 0.14 mmol) was placed in a flame-dried round-bottom flask, followed by the addition of sodium hydride (NaH, 1 mg, 0.042 mmol) and dry dioxane (1.0 mL) or dry DMSO (0.2 mL) to generate high- or low-molecular-weight hbPG, respectively.[4] The mixture was stirred at 95 °C under an argon atmosphere for 30 min. Glycidol (1.0 mL, ≈1.1 g, 14.8 mmol) was then added slowly at a rate of 0.04 mL h^{-1} using a syringe pump. After completion, the reaction was quenched with 0.1 N HCl in methanol and stirred for 1 h. The polymer was solubilized in methanol and precipitated into chilled diethyl ether, dialyzed against water (MWCO 1 kDa), and lyophilized.

Table S4. Characterization of hbPGs obtained from TMP as initiator.

**hbPG**	**M_n (kDa)[a]**	**Đ[a]**	**Relative abundance (%)[b]**						**DB[b]**
			$L_{1,3}$	D	$L_{1,4}$	T_1	T_2	diol	
$hbPG_{12k}$	12.1	2.5	12	24	27	37	0	51	0.55
$hbPG_{50k}$	49.8	1.3	11	30	29	32	0	44	0.60

[a]Determined by GPC-RI-MALS in DMF (+LiBr 0.1%). [b]Determined by inverse-gated ^{13}C NMR.

Synthesis of nanogel cross-linked with Bz-2B: The nanogels were prepared from $hbPG_{12k}$ and $hbPG_{50k}$. Bz-2B was first dissolved at a concentration of 1.12 mg/mL in the carbonate buffer containing 10% DMSO, and the polymer was dissolved at a concentration of 4 mg/mL in the carbonate buffer. Then, 1 mL of Bz-2B was added at a rate of 0.04 mL/h over 1 mL of the polymer solution. The resulting nanogel was diluted to generate four distinct polymer concentrations ranging from 2 to 0.7 mg/mL. As a reference, polymer solutions were prepared at identical concentration range in the carbonate buffer with 5% DMSO.

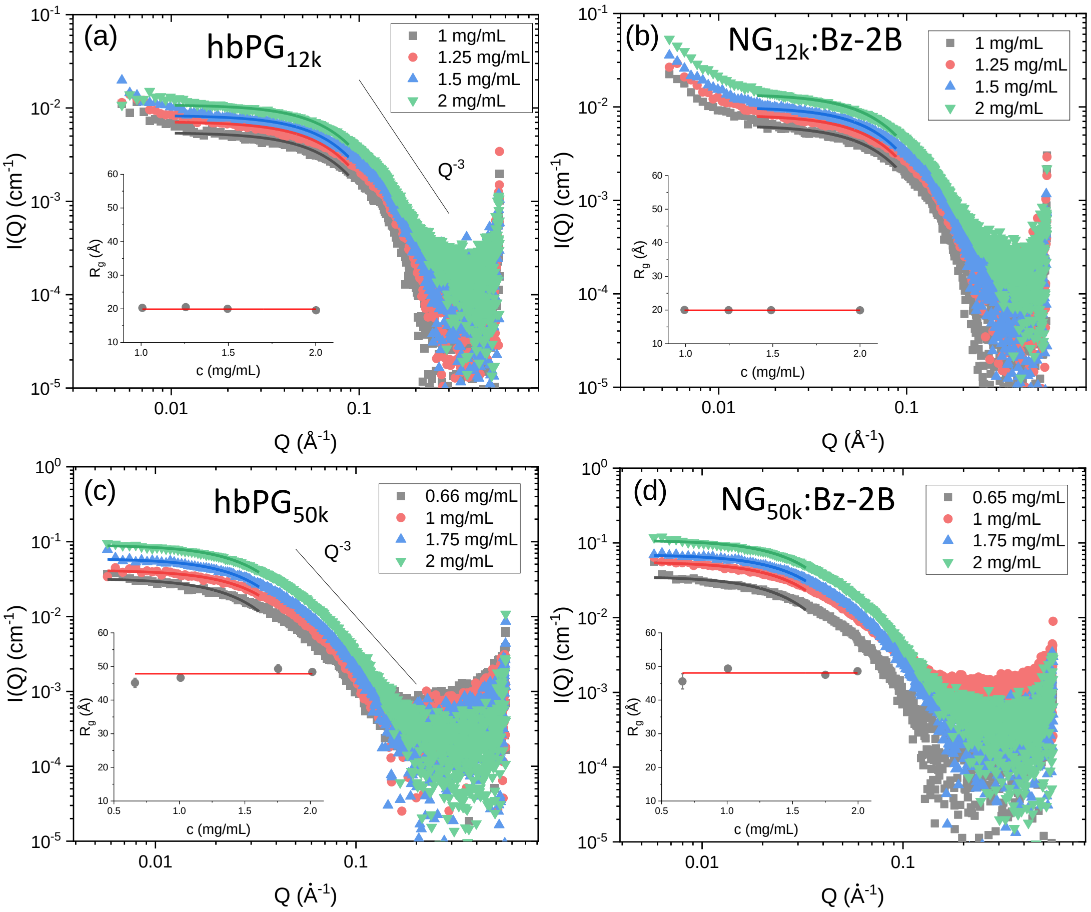

Figure S10. SAXS profiles of $hbPG_{12k}$ and $hbPG_{50k}$ precursors and the corresponding nanogels at different concentrations (1–2 mg mL^{-1}) in carbonate buffer of pH 9. Panels (a) and (c) show the $hbPG_{12k}$ and $hbPG_{50k}$ precursors, while panels (b and d) show the corresponding nanogels. Symbols correspond to experimental data at different concentrations, as indicated in the legends. Solid lines represent Guinier fits applied in the low-Q regime. Insets display the resulting radius of gyration (R_g) as a function of concentration. The red horizontal line denotes the average R_g value, highlighting the absence of significant concentration dependence. The solid gray lines correspond to a scaling of $\sim Q^{-3}$ (compact structures) and serve as references.

Table S5. Results from Guinier analysis of hbPG and their nanogels.

Entry	Structure	M_w (kDa)	R_g (Å)
6	$hbPG_{12k}$	7.5 ± 0.1	19.9 ± 0.2
6	NG_{12k}:Bz-2B	10.1 ± 0.2	20.0 ± 0.1
7	$hbPG_{50k}$	58 ± 3	47.8 ± 0.7
7	NG_{50k}:Bz-2B	67 ± 4	48.1 ± 0.5

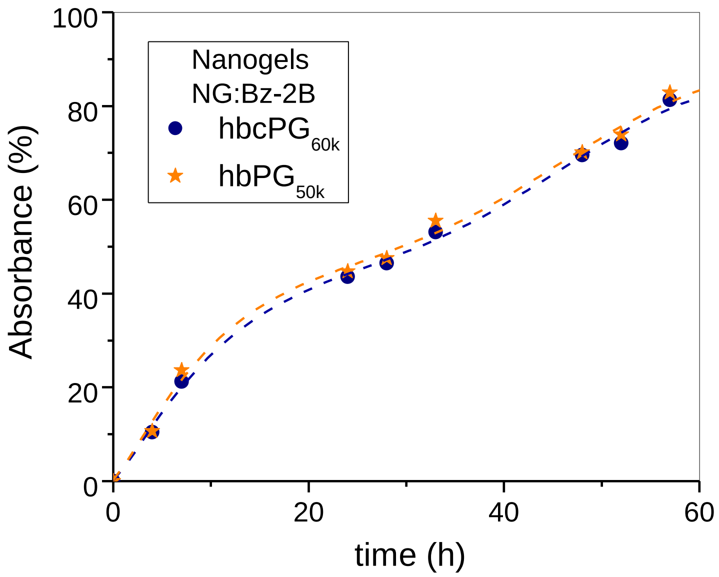

Figure S11. Comparison of RhB release from NG:Bz-2B derived from $hbPG_{50k}$ and $hbcPG_{60k}$ in buffer at pH 9 at [boronic acid] / [diol] molar equivalent ratio = 0.5. Dashed lines correspond to fits obtained using Equation 7. The release profiles are faster than those shown in Figure 9 due to the use of a U-shaped glass setup (see Figure 1) equipped with a larger diameter membrane (3 cm compared to 1 cm in Figure 9), thereby increasing the diffusion area for RhB between the [S] and [M] compartments.

References

1. Gómez Urreizti, E.; Gastearena, X.; Lam, A.; González de San Román, E.; Miranda, J. I.; Matxain, J. M.; Barroso-Bujans, F., Kinetics of heterogeneous polymerization of glycidol with B(C6F5)3 in toluene in the absence and presence of water. *Mater. Today Chem.* **2024,** 37, 101993.
2. Al Assiri, M. A.; Gómez Urreizti, E.; Pagnacco, C. A.; Gónzalez de San Román, E.; Barroso-Bujans, F., Reactivity of B(C6F5)3 towards glycidol: The formation of branched cyclic polyglycidol structures. *Eur. Polym. J.* **2022,** 171, 111194.
3. Sunder, A.; Hanselmann, R.; Frey, H.; Mülhaupt, R., Controlled Synthesis of Hyperbranched Polyglycerols by Ring-Opening Multibranching Polymerization. *Macromolecules* **1999,** 32, (13), 4240-4246.

4. Pagnacco, C. A.; Alvarez-Fernandez, A.; Maestro, A.; González de San Román, E.; Lund, R.; Barroso-Bujans, F., Varying the Core Topology in All-Glycidol Hyperbranched Polyglycerols: Synthesis and Physical Characterization. *Macromol. Rapid Commun.* **2025,** 46, 2400791.
